# Aquatic islands in the sky: 100 years of research on water‐filled tree holes

**DOI:** 10.1002/ece3.9206

**Published:** 2022-08-12

**Authors:** Jana S. Petermann, Martin M. Gossner

**Affiliations:** ^1^ Department of Environment and Biodiversity University of Salzburg Salzburg Austria; ^2^ Forest Entomology Swiss Federal Research Institute WSL Birmensdorf Switzerland; ^3^ ETH Zurich, Department of Environmental Systems Science Institute of Terrestrial Ecosystems Zurich Switzerland

**Keywords:** aquatic insects, container habitat, detritus, food web, metacommunity, phytotelm

## Abstract

Water‐filled tree holes are unique ecosystems that may occur high up in tree crowns and are essentially aquatic islands in the sky. Insect larvae, mesofauna, and other organisms colonize the waterbodies and feed on the accumulating detritus. Water‐filled tree holes are not only important habitats for these species but have been used as model systems in ecology. Here, we review more than 100 years of research on tree‐hole inhabiting organisms and show that most studies focus on selected or even single species (most of which are mosquitoes), whereas only few studies examine groups other than insects, especially in the tropics. Using a vote counting of results and a meta‐analysis of community studies, we show that the effects of tree‐hole size and resources on abundance and richness were investigated most frequently. Both were found to have a positive effect, but effect sizes were modulated by site‐specific environmental variables such as temperature or precipitation. We also show that parameters such as the height of the tree holes above ground, tree‐hole density, predation, and detritus type can be important drivers of organism abundance or richness but are less often tested. We identify several important research gaps and potential avenues for future research. Specifically, future studies should investigate the structure, functions, and temporal dynamics of tree‐hole food webs and their cross‐system interactions, for example, with terrestrial predators that act as a connection to their terrestrial surroundings in meta‐ecosystems. Global observational or experimental tree‐hole studies could contribute pivotal information on spatial variation of community structure and environmental drivers of community assembly. With a better understanding of these unique aquatic habitats in terrestrial ecosystems, natural and artificial tree holes can not only serve as model systems for addressing fundamental ecological questions but also serve as indicator systems of the impacts of environmental change on ecosystems.

## Graphical abstract

Water‐filled tree holes are unique ecosystems that are inhabited by insect larvae, mesofauna and other organisms. In our review and meta‐analysis we show that most studies on tree‐hole inhabitants focus on selected or even single species, and that the few community studies consistently found positive effects of tree‐hole size and resources on abundance and richness of inhabitants, sometimes modulated by environmental parameters such as annual temperature. We identify potential avenues for future research and argue that a better understanding of water‐filled tree holes will facilitate their use as model systems for addressing fundamental ecological questions and as indicator systems of the impacts of environmental change on ecosystems.

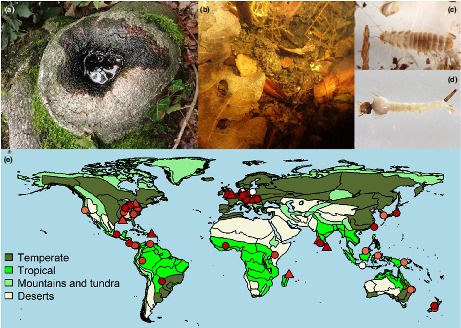

## 1 INTRODUCTION

Very few biotic frontiers remain on this planet. The canopies of forests have been identified as one of these last frontiers of ecological knowledge (Erwin, 1983). We know very little about the organisms that live up there and what the specific environmental conditions are that may structure their communities. Water‐filled tree holes are a very special habitat in forest canopies and have fascinated ecologists for a long time, with first reports on these habitats and their inhabitants dating back more than 100 years (Christophers & Chand, 1916; Müller & Müller, 1878). Although water‐filled tree holes occur also at the base of trees or in deadwood, the number of canopy holes (>2 m above ground, Kitching, 1971) per hectare can be significantly higher than the number of ground holes (Gossner, Lade, et al., 2016). These water‐filled tree holes in the canopy have been termed—together with other similar systems such as bromeliads—“hanging aquaria” (Brehm, 1925; Maguire, 1971; Müller & Müller, 1878) and constitute aquatic islands in the sky, being more or less isolated from other water sources but still hosting a large number of aquatic and semi‐aquatic species such as protozoans, rotifers, nematodes, insects, crustaceans, and amphibians, but also algae, fungi, and bacteria (Figure S1; for species lists of tree‐hole inhabitants see Thienemann, 1934, Rohnert, 1950, Kitching, 2000, Greeney, 2001). Tree holes form as rot holes from branch breaks or wood pecker holes, or as pan holes in branch axils or stem forks (Figure S2). They can also form close to the ground in cut tree trunks (rot holes) or buttress roots (pan holes). Tree holes can be quite abundant in forests. Studies have found up to 56 tree holes/ha with a total of 45 L of water in German forests (Gossner, Lade, et al., 2016).

The communities that assemble when these holes fill up with water depend largely on dead organic matter (mostly leaf litter) accumulating in the holes (Kitching, 1987b; Pimm & Kitching, 1987) and have also been shown to be affected by nutrient and chemical input of stemflow water (e.g., Carpenter, 1982). The detritus constitutes the most essential resource for species in tree holes that are largely detritivores, and decomposition is thus the most important ecosystem function in these systems. The communities of water‐filled tree holes may experience harsh and very dynamic conditions, especially in temperate areas, with frequent drought and freezing events (Gossner, 2018). In tropical rainforests, tree holes may be much more benign habitats (Pimm & Kitching, 1987). Many species depend on water‐filled tree holes for their larval stages (e.g., insects such as beetles and dipterans or amphibians), their entire life cycles (e.g., nematodes and other mesofauna), or as important water source (Kirsch et al., 2021), especially where very little other open water exists. Communities in tree holes can vary strongly between holes in the canopy vs. lower forest strata (Blakely & Didham, 2010; Gossner & Petermann, 2022; Yanoviak, 1999b). They seem to be distinct from communities in other habitats (Blakely et al., 2012) and some species may even be more or less exclusive to tree holes, for example several detritivorous (Kitching, 1971) and predatory (Yanoviak, 1999a) insect species (Rohnert, 1950; Yanoviak, 2001a), but also tree frogs (Inger, 1966; Lardner & bin Lakim, 2002; Strauß et al., 2018). Blakely et al. (2012) showed for temperate rainforests of New Zealand that <10% of the species in this study used tree holes and adjacent ground‐based freshwater bodies as habitat while 18% of the species were actually exclusive to tree holes. Other species seem to be generalist aquatic decomposers such as aquatic hyphomycetes in a tropical forest in the Western Ghats, India, with 17 of 18 species occurring in both tree holes and streams, albeit with different relative abundances (Sridhar et al., 2013). A further microscopic organism group—algae—were thought to be present only in very low numbers in tree holes (Kaufman et al., 2001), but a recent study found the opposite (Ptatscheck & Traunspurger, 2015). Bacteria and fungi are likely also important parts of the nutrient cycles in tree holes, but very little information exists on these groups (but see Gönczöl & Révay, 2003; Magyar et al., 2017; Sridhar et al., 2013; Verdonschot et al., 2008). Even many macroscopic species that inhabit tree holes have not been identified because they are only found in larval stages and only few experts exist, especially for tropical insects. Recently, some species new to science were found in tree holes (e.g., Grinang et al., 2015; Polhemus, 1999). Even if individuals can be identified to species level, little is known on their life history traits, their dispersal ability, and feeding ecology. Interestingly, there seem to be few obligate macroscopic predators in European tree holes (Schmidl et al., 2008; Tate, 1935), but some species may be facultative predators or scavengers. In other temperate forest such as in the east and south of North America (Griswold & Lounibos, 2006) and in particular in tropical forests (Fincke et al., 1997), predatory insects or vertebrates seem to be more important and top‐down control a more likely mechanism in structuring the communities (Kitching, 2000).

It has been recognized that water‐filled tree holes constitute natural microcosms that can be useful model systems (Srivastava et al., 2004). For example, they have been used to study productivity–richness relationships (Srivastava & Lawton, 1998) and local–global richness patterns (Srivastava, 2005). Water‐filled tree holes are likely organized as metacommunities with local communities being connected by limited dispersal (Leibold et al., 2004) and could be employed in tests of metacommunity concepts (Ellis et al., 2006). Water‐filled tree holes do interact with the canopies and other terrestrial habitats around them (Kitching, 1971), for example, through receiving leaf litter as a subsidy and providing emerging insects as a reverse subsidy. Thus, they form ideal study systems in which aquatic and terrestrial environments are tightly integrated and allow meta‐ecosystem approaches (Scherer‐Lorenzen et al., 2022; Soininen et al., 2015) on small scales, something that is more difficult for larger systems such as lakes or streams and their terrestrial environment. Tree holes have been found to be affected by anthropogenic environmental alterations such as forest management or pollution (Ager et al., 2010; Gossner, Lewinsohn, et al., 2016; Petermann et al., 2016; Petermann et al., 2020; Yanoviak et al., 2006) and could potentially be used as indicators of environmental change. Conversely, they can themselves affect human health by providing breeding grounds for mosquito species that carry diseases. There have been many medically motivated studies in the tropics and subtropics censusing certain disease‐carrying mosquito species in these systems (e.g., Carlson et al., 2004; Hoshi et al., 2014; Li et al., 2014; Mangudo et al., 2014; Mangudo et al., 2015; O'Meara et al., 1993; Omlin et al., 2007; Walker et al., 1996; Weterings et al., 2014). With a changing climate and increased global transportation, tree‐hole breeding mosquitoes acting as disease vectors could become a more significant concern in temperate areas as well (Cebrián‐Camisón et al., 2020).

Several articles and books have reviewed studies on the communities in different types of plant‐held waters or “phytotelmata,” including tree holes. Early studies summarized information on species occurrences in different types of tropical phytotelmata (Thienemann, 1934). Maguire (1971) reviewed research on processes that may structure phytotelm communities, such as in bromeliads and pitcher plants, including their artificial analogs. He almost exclusively focused on protozoans and small metazoans and reports on experiments, especially on colonization processes, done in artificial systems (bottles and beakers) in the field. Kitching (2000, 2001) summarized the work that was done on tree holes and other natural “container habitats” up to 1997 with a special focus on food webs, drawing fundamental conclusions on food web structure, and evolution from phytotelm research. Greeney (2001) provided data on the occurrence of aquatic and semi‐aquatic insect taxa in different plant‐held waters, including tree holes. A more recent article by Nishadh and Das (2014) qualitatively summarized selected tree‐hole studies between 1950 and 2013 to describe inhabitants, processes, and interactions and concluded that more research on these habitats needs to be done in Tropical Asia.

To complement these previous studies, we here exhaustively review more than a 100 years of global water‐filled tree‐hole research and conduct a vote counting and a meta‐analysis on certain aspects of tree‐hole community ecology. Our aim is to qualitatively and quantitatively summarize the knowledge on tree‐hole communities and specifically their environmental drivers, as well as to identify knowledge gaps and avenues for future research on water‐filled tree holes.

## 2 METHODS

We used Web of Science (http://apps.webofknowledge.com) for a thorough search for tree‐hole studies (last update on 02.11.2021). The following databases were included: Web of Science Core Collection (1900‐present), BIOSIS Citation Index (1926‐present), BIOSIS Previews (1969‐present), Current Contents Connect (1998‐present), Data Citation Index (1900‐present), KCI‐Korean Journal Database (1980‐present), MEDLINE® (1950‐present), Russian Science Citation Index (2005‐present), SciELO Citation Index (1997‐present), and Zoological Record (1864‐present). The following search string was used on topic fields (titles, abstracts, keywords, and indexing fields such as systematics, taxonomic terms, and descriptors) to include as many aquatic tree‐hole studies as possible while excluding studies on nesting birds, ornamental plants or transport: TS = (tree‐hole* OR treehole* OR “tree hole”* OR phytotelm* OR dendrotelm* OR “tree cavity”*) AND TS = (tree* AND [water* OR aquatic*]) NOT TS = (nest‐box* OR “nest box”* OR nursery* OR ornamental* OR horticult* OR seedling* OR transport* OR containerized*). Our search yielded 1134 publications (Figure S3). Seventy‐five empirical research articles were added from our own databases that were not found by Web of Science for various reasons (e.g., abstract, keywords, or title did not contain our keywords, article was in German). We also added six own unpublished data sets. We then scanned title (and if necessary: abstract), deleted double records, and excluded those publications that described patents or merely reported about equipment or methods (mostly of mosquito control). We also excluded those records that dealt with water bodies other than tree holes (bromeliads, pitcher plants, ferns, *Pandanus* leaves, bamboo stumps, *Dipsacus*, inflorescences, nuts, ponds, rivers, rock pools, rice fields, tires, and various artificial containers unless these other types of water bodies were explicitly used as mimics of tree holes), were purely medical or social studies, described the use of tree holes (dry and wet) by terrestrial organisms or abiotic effects only (no organisms sampled).

From the remaining 932 publications on water‐filled tree holes, we only retained primary research articles and excluded book chapters, comments and editorials, meeting abstracts and workshop proceedings, identification keys, species descriptions, species distributions and inventories, and reviews (the most relevant reviews are described in the introduction).

We report general findings from this literature search, with a focus on community studies in the form of a qualitative and quantitative review. Community studies were defined as those that investigate all species of certain organism groups, even if they do not study all organisms in the tree hole. The groups that we used here were based on species that were commonly studied together even if they are not necessarily delineated on a taxonomic basis: insects, mites, polychaetes, gastropods, microcrustaceans, crabs, amphibians, protists, bacteria, fungi, nematodes, rotifers, and tardigrades. We included studies on natural tree holes and studies on artificial tree‐hole analogs if they specifically referred to tree holes. We included observational studies and experiments. Experiments were defined as those studies that actively manipulated at least one variable and tested the effect on the tree‐hole community. Thus, studies on artificial tree holes were classified as observational studies if no variable was manipulated and/or tested.

In addition to the review, we conducted a vote counting of results reported in community studies for the most important effects of environmental variables on tree‐hole organisms (overall abundance and richness). Results from different organism groups or different explanatory or response variables reported from one study were included as separate responses in the vote counting. Unfortunately, there were not enough studies to analyze effects on any other response variables besides abundance and richness (such as e.g., community composition or functions such as decomposition). We also ran formal meta‐analyses for those effects that we could gather sufficient data for, that is, the effect of tree‐hole size (maximum or actual water capacity) on the abundance and richness of tree‐hole fauna and the effect of detritus amount (weight or volume) on tree‐hole communities. For the meta‐analyses, we compiled raw data from unpublished or published community studies where possible. If data were available as a figure only, we used the Web‐based tool WebPlotDigitizer (https://automeris.io/WebPlotDigitizer/) to extract the data points. We also used Pearson's correlation coefficients (*r*) from published studies reporting the effects of tree‐hole size or detritus amount on the abundance and richness of organisms. Furthermore, *t*, *F*, and χ^2^ values were converted to correlation coefficients in some instances. Correlation coefficients between tree‐hole size or detritus amount, respectively, and organism abundance or richness, respectively, were then used as effect sizes in four meta‐analyses after transformation to Fisher's *Z*. We ran additional analyses using partial correlation coefficients for those studies for which we had raw data on both detritus amount and size to control for the effects of size on detritus amount. Data on separate groups of organisms or response variables from one study were used as separate data points. We first ran a random effects model, treating heterogeneity among studies as purely random and not using moderators (Viechtbauer, 2010), for each combination of explanatory variables (size or detritus) and response variable (abundance or richness) to assess the overall effects across all studies. Funnel plots and regression tests for asymmetry were run to test for publication bias, and the “trim‐and‐fill” method was applied in case of a bias (Viechtbauer, 2010). We then used annual mean temperature (in °C) and annual precipitation (in mm) as well as other site or study characteristics (latitude, longitude, natural vs. artificial tree holes, insects only vs. other organisms besides insects included) as moderators in mixed‐effects meta‐analyses (Viechtbauer, 2010). Annual mean temperature and annual precipitation were used as reported by the studies. If this information was not available from the publication, temperature and precipitation data were extracted from the WorldClim database (version 1.4, www.worldclim.org). For those studies that used several sites, averages of the climate data between the sites were used. If several sites per study were used, these were never more than 300 km apart and climates did not differ much. Meta‐analyses were conducted with the metafor package version 2.1–0 (Viechtbauer, 2010) in R version 3.6.2 (R Development Core Team, 2016).

## 3 RESULTS

### 3.1 Organisms

**FIGURE 1:**
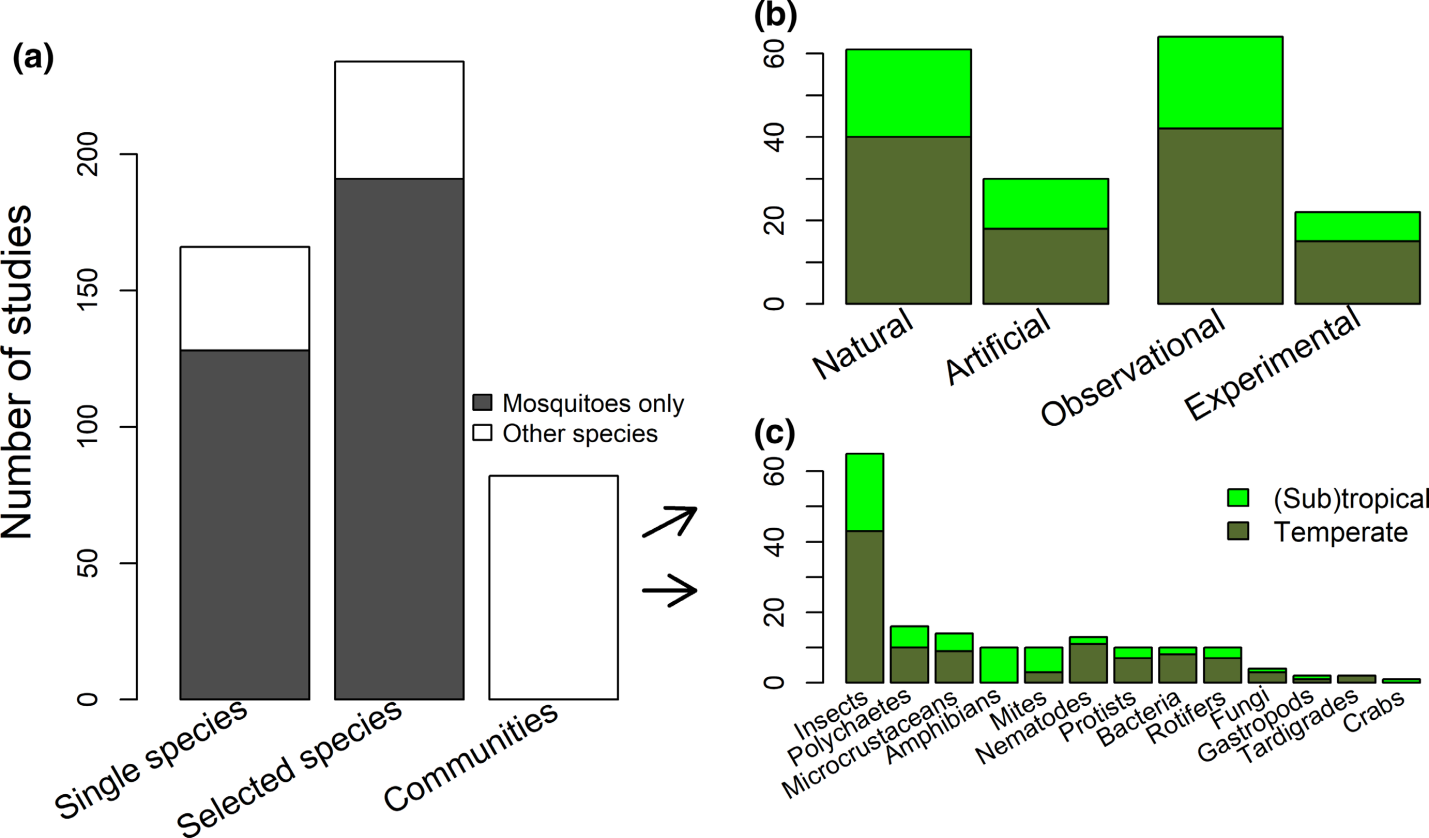
(a) Numbers of research articles on tree holes with a field component (total *n* = 482) reporting results on single species, multiple selected species or communities (“communities” were defined as groups listed under c). Studies dealing with mosquito species only are shaded in dark gray, all other studies are presented in white. (b) Number of community studies (*n* = 82) which included natural or artificial tree holes (10 studies included both types) and which had observational or experimental parts (five had experimental and observational parts). “Experimental” studies were defined as those which specifically manipulated abiotic or biotic variables. Therefore, studies in artificial tree holes could also be observational if they did not manipulate any additional variables. (c) Number of community studies (*n* = 82) reporting results on the different groups. About half of these studies (*n* = 39) reported results for more than one of these groups. Bars in (b,c) are split up into studies in temperate sites (dark green) and studies in tropical sites (light green).

Our search and classification procedure yielded 653 empirical research articles on organisms from water‐filled tree holes (Figure S3). Of these, 161 were pure laboratory studies on tree‐hole‐associated species. Of the remaining 497 studies that at least collected some data in the field, 15 were done on artificial containers that were not used to mimic tree holes (i.e., not “artificial tree holes”). Of the remaining 482 studies on natural and artificial tree holes, 234 studies sampled a limited or selected set of species from a particular organism group, that is, more than one but not all species of that group (groups of organisms were as follows: insects, mites, polychaetes, gastropods, microcrustaceans, crabs, amphibians, protists, bacteria, fungi, nematodes, rotifers, and tardigrades). In addition, 166 studies even only studied a single species (Figure 1a). The studies covering only limited sets of species and the single‐species studies largely focused on mosquitoes (191 of 234 limited‐species studies and 128 of 166 single‐species studies, respectively), often with a particular interest in mosquitoes as vectors of certain diseases.

**FIGURE 2:**
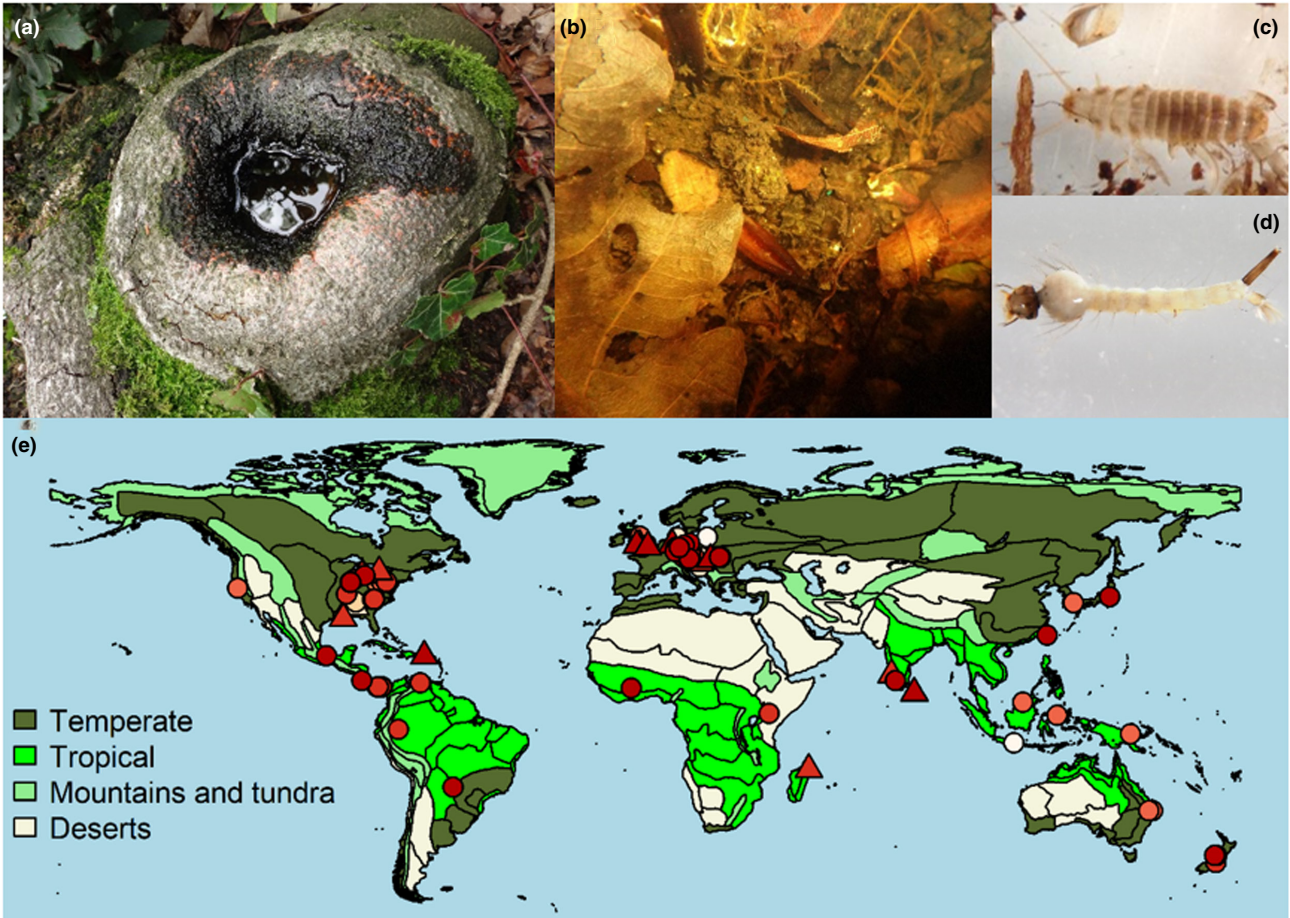
(a) Water‐filled tree‐hole ecosystem, (b) water and detritus accumulating in the tree hole, (c) specialist beetle species inhabiting European tree holes: Larval *Prionocyphon serricornis*, (d) generalist mosquito species inhabiting European tree holes: Larval *Culex pipiens*, (e) global map depicting biomes (summarized from Udvardy, 1975) and the study sites of community studies (one site per study shown, only few studies used more than one site and these were usually close together). The more recent the study the darker red the symbol. Studies including insects are shown with circles, studies on other organisms with triangles. Photographs: J. S. Petermann.

The remaining part of this article only examines those studies that included entire communities of certain organism groups (e.g., entire insect communities), even if they did not study all organisms in the tree hole (total *n* = 82 studies, Table S1). Of all community studies, 62 included natural tree holes (Figure 1b, 21 [sub]tropical, 40 temperate, and one with a temperate and a subtropical field site) and 30 included artificial tree‐hole analogs (12 [sub]tropical and 18 temperate). A total of 60 community studies included observational studies (20 [sub]tropical, 39 temperate, and one with a temperate and a subtropical field site), 22 included experiments, manipulating at least one variable (7 [sub]tropical and 15 temperate). Of all community studies, the majority included insect communities (*n* = 66 studies, Figure 1c). However, other groups were also investigated in a number of studies, such as mites (e.g., Devetter, 2004; Jenkins et al., 1992), polychaetes (e.g., Schulz et al., 2012), gastropods (e.g., Kitching, 1987a), microcrustaceans (e.g., Devetter, 2004; Schulz et al., 2012), crabs (e.g., Cumberlidge et al., 2005), amphibians (e.g., Pimm & Kitching, 1987; Yanoviak, 2001a, 2001b), protists (e.g., Walker et al., 2010; Yee et al., 2007), bacteria (e.g., Ager et al., 2010; Bell et al., 2005; Kaufman et al., 2008; Petermann et al., 2020; Ponnusamy et al., 2008; Verdonschot et al., 2008; Walker et al., 2010; Woodcock et al., 2007), fungi (e.g., Gönczöl & Révay, 2004; Kaufman et al., 2008; Magyar et al., 2017; Sridhar et al., 2013), nematodes (e.g., Devetter, 2004; Petermann et al., 2020; Ptatscheck & Traunspurger, 2014; Ptatscheck & Traunspurger, 2015), rotifers (e.g., Devetter, 2004; Ptatscheck & Traunspurger, 2014; Ptatscheck & Traunspurger, 2015), tardigrades (e.g., Devetter, 2004; Ptatscheck & Traunspurger, 2014), and algae (e.g., Ptatscheck & Traunspurger, 2015). Studies on amphibians were almost exclusively done in the tropics as frogs are only rarely reported from tree holes in temperate areas (but see e.g., von Brandt, 1934; Kirsch et al., 2021) and are not known to breed in tree holes there (but see Nöllert, 2012 for a single instance of a Rana t. temporaria brood in a lying dead beech tree). We also found more studies on mites from the tropics than from temperate areas, where aquatic mites seem to be rarely occurring in tree holes (or may have been overlooked), while tree‐hole nematodes, protists, bacteria, and rotifers are studied more often in temperate areas. Most community studies took place in Europe and North America, and those studies also regularly examined other organisms than insects (Figure 2). Many areas worldwide have no or few tree‐hole community studies, and the few studies in areas other than Europe and North America mostly report results on insect communities only.

### 3.2 Environmental effects on tree‐hole communities

**FIGURE 3:**
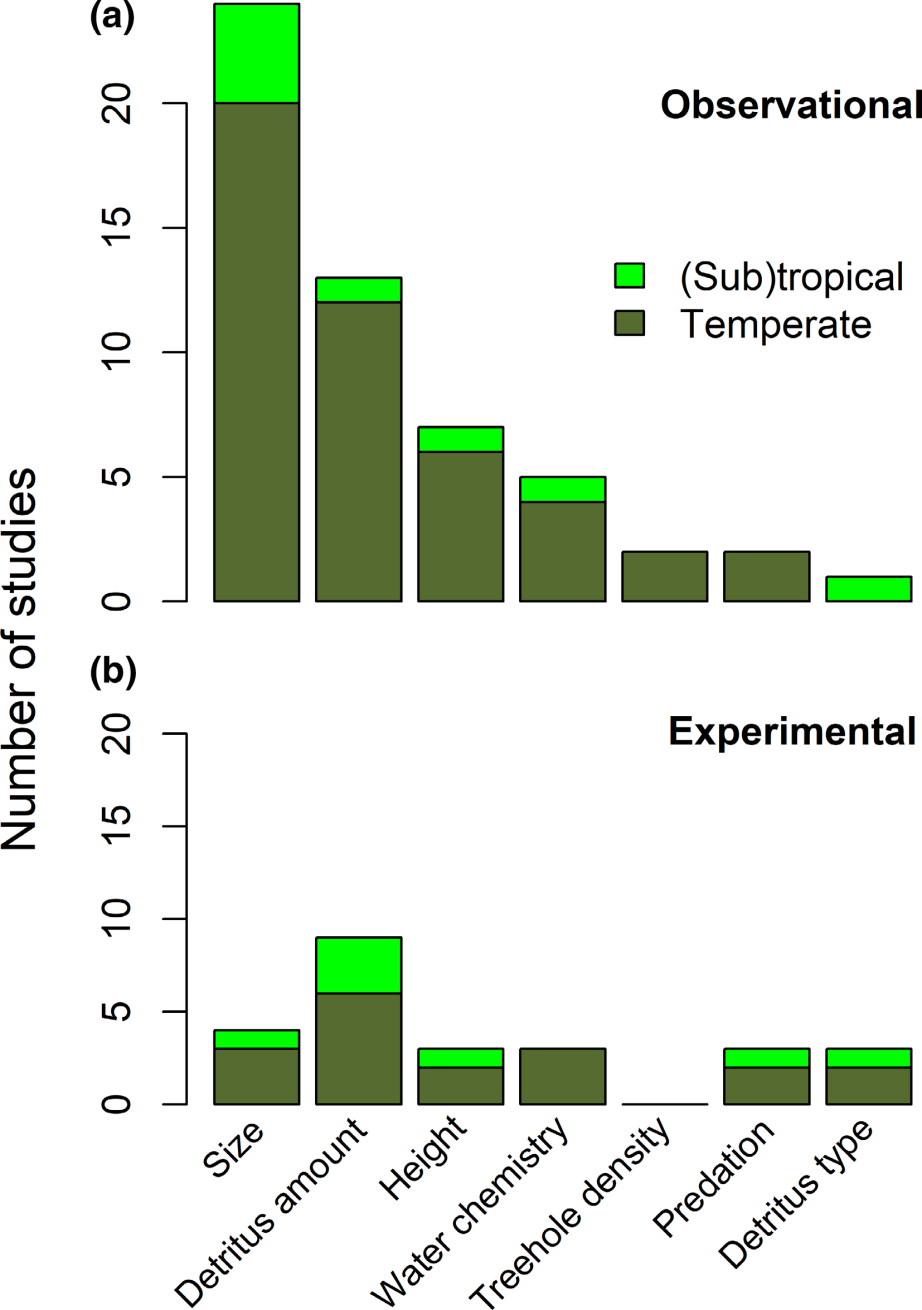
(a) Numbers of observational community studies and the abiotic and biotic environmental variables that were most often measured and analyzed (i.e., the effect on organism abundance or richness was tested). (b) Numbers of experimental community studies and the environmental variables they manipulated. “Experimental” studies were defined as those which specifically manipulated abiotic or biotic explanatory variables. Therefore, studies in artificial tree holes were counted as observational studies if they did not manipulate these variables. Some studies included both observational and experimental approaches or addressed several explanatory variables and thus, are counted several times. Bars are split up into studies in temperate sites (dark green) and studies in tropical sites (light green). Size was measured or manipulated as potential volume when full (maximum capacity) in 12 studies, as actual volume in 17 studies and as surface area in 2 studies. Detritus type may refer to animal vs. plant detritus or different species of plant detritus.

**FIGURE 4:**
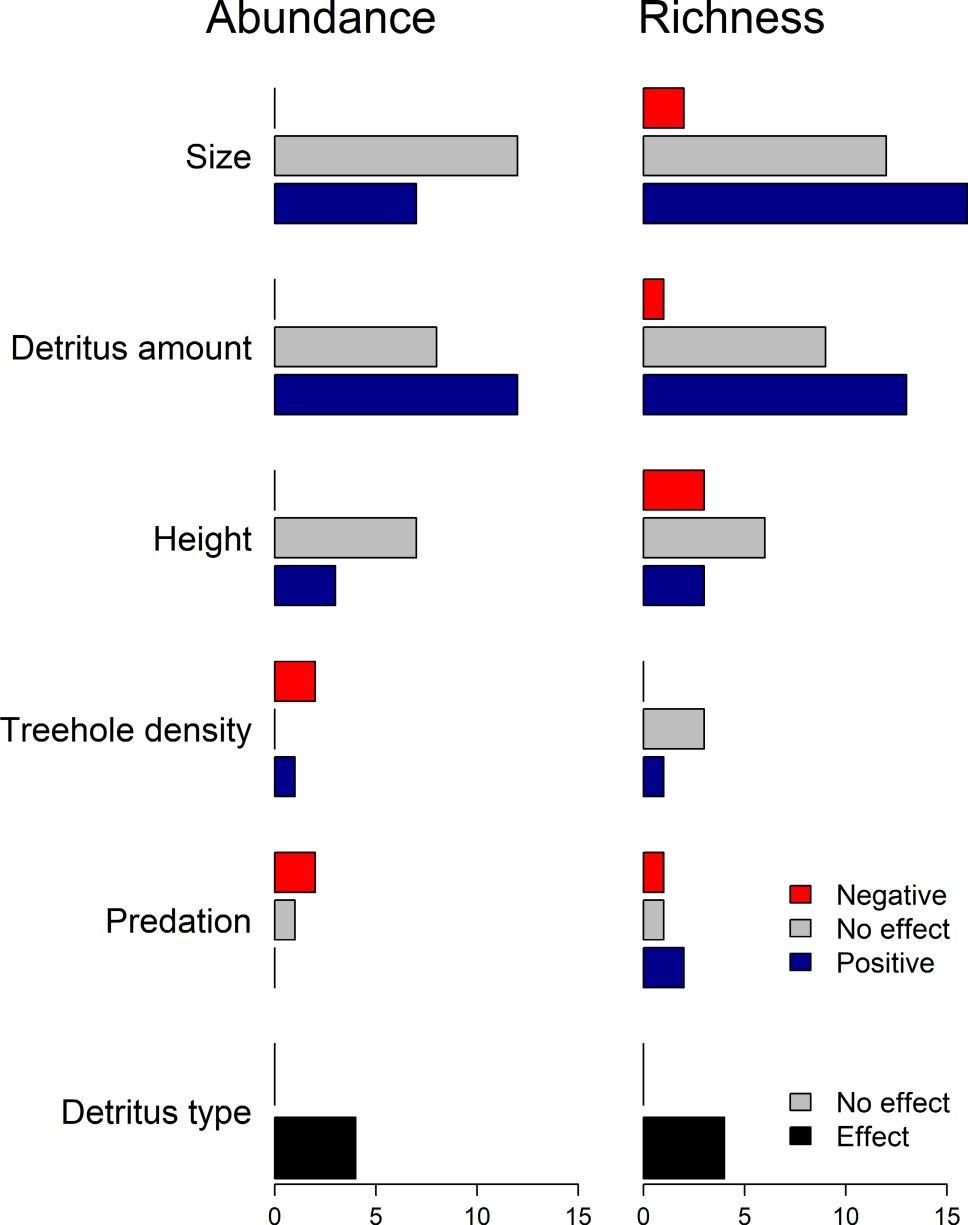
Vote counting of effects of abiotic and biotic variables on the abundance and species richness of tree‐hole communities. Both observational and experimental studies were included and negative effects (red), nonsignificant effects (gray), and positive effects (blue) reported by these studies were recorded. Some studies addressed several explanatory variables and appear several times for these separate variables. Studies were also counted several times if they investigated separate organism groups (as defined and listed in Figure 1c). Water chemistry does not appear here because the various variables that were investigated in this context could not be presented in a simplified way. Abundance was most often assessed as raw abundance per tree hole (*n* = 13 observational, *n* = 9 experimental). Only in a few studies, abundance was assessed as density (i.e., per water volume, *n* = 4 observational, *n* = 3 experimental). Two of the observational studies used raw abundance for insects and density for other organisms (protists and nematodes, respectively). Detritus type may refer to animal vs. plant detritus or different species of plant detritus and nonsignificant differences between detritus types are shown in gray, significant differences in black.

**FIGURE 5:**
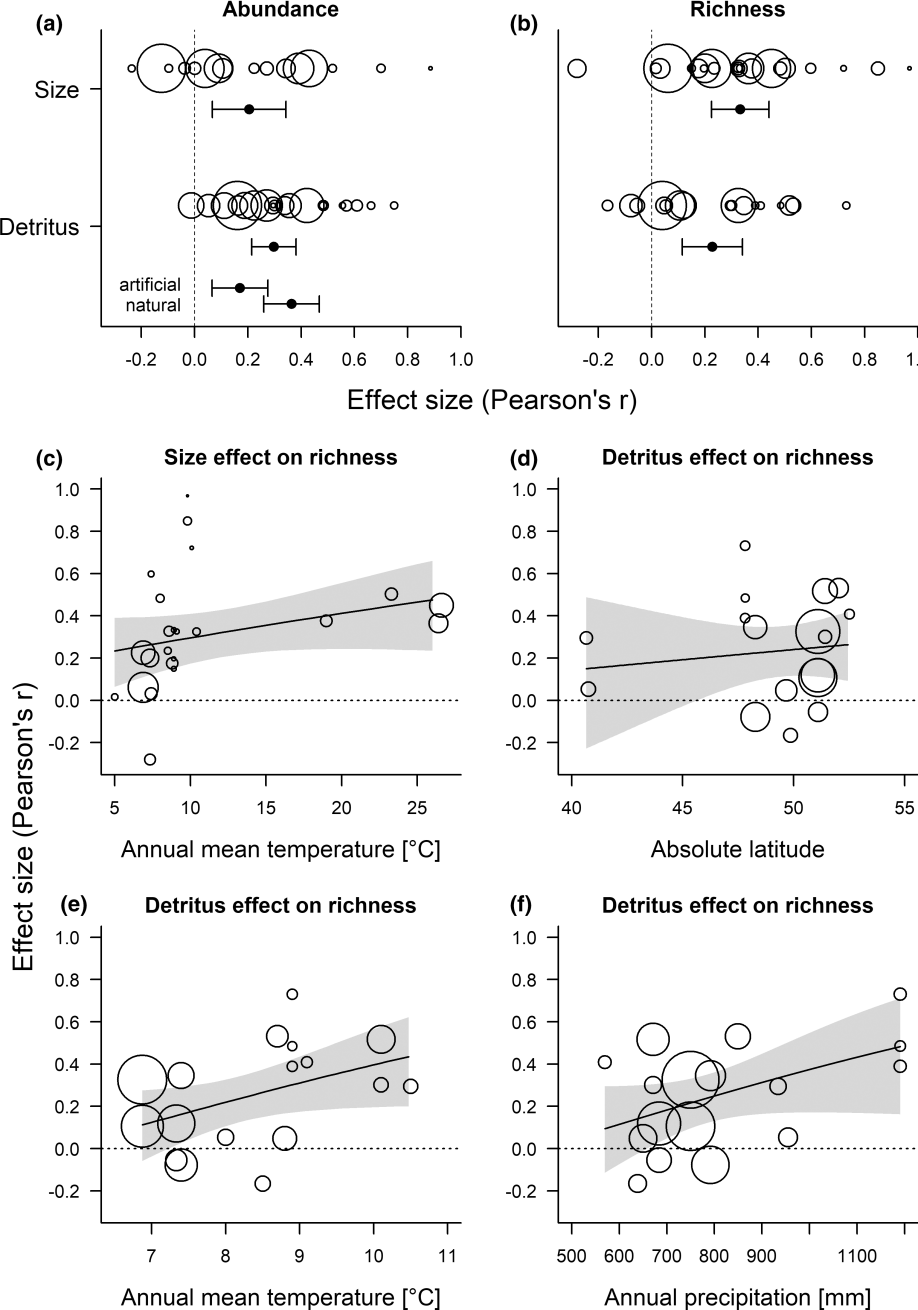
Results of random effects meta‐analysis of the effect of tree‐hole size (actual or maximum volume) and of the effect of detritus amount (weight or volume) on (a) abundance and (b) richness of tree‐hole organisms. Shown are estimates and 95% confidence intervals from the models (Pearson's *r*, backtransformed from Fisher's *Z*), as well as predicted values for each study scaled to be proportional to their inverse standard errors. In all cases, the overall effects of size and detritus amount were positive and significant. Additional estimates in a are from a separate models showing the significant difference of the effect of detritus on abundance between artificial and natural tree holes. (c–f) show selected significant effects of moderators in mixed‐effects meta‐analyses using regression lines and 95% confidence intervals from the models (see Table S2 for results of models). (c) Stronger effects of tree‐hole size on organism richness were found under higher annual mean temperature. Stronger effects of detritus amount on organism richness were found (d) at higher absolute latitudes, (e) under higher annual mean temperature and (f) under higher annual precipitation. One study with very high annual mean temperatures and high annual precipitation from a low latitude was excluded from D‐F. the models gave similar results with and without this outlier. Size was measured as maximum volume (a: *N* = 7, b,c: *N* = 11) or actual volume (a: *N* = 9, b,c: *N* = 12). One study using surface area as a proxy of size was excluded here. Abundance (a) was most often assessed as raw abundance per tree hole (size: *N* = 14, detritus: *N* = 17). Only in a few studies, abundance was assessed as density (i.e., per water volume, size: *N* = 2, detritus: *N* = 2). One study of detritus assessed insects in terms of their abundance and protists in terms of density.

The effects of environmental variables on tree‐hole communities are a common topic in many studies. Most frequently, the water volume of the tree hole or related measures of tree‐hole size are investigated. Size‐related variables are considered important since they are a proxy of drought risk, especially in combination with high temperatures or strong exposure to the sun, for example, higher up in trees (see below). Thus, 25 observational setups and four experiments addressed the effect of tree‐hole size (Figure 3). Size was measured or manipulated as potential volume when full (maximum capacity) in 12 studies, as actual volume in 17 studies and as surface area in 2 studies. Many studies found strong effects of tree‐hole volume (or other size‐related measures) on tree‐hole communities, with higher abundance, richness, and different community composition in larger holes (Figure 4 and Figure S4). Our meta‐analyses showed that the effects of tree‐hole size on abundance (df = 15, *Z* = 2.81, *p* = .005, Figure 5a) and richness (df = 22, *Z* = 5.58, *p* < .001, Figure 5b) were overall positive and significant. Funnel plots (Figure S6) and regression tests for asymmetry indicated a marginally significant publication bias only in the effect of size on richness, and thus, the “trim‐and‐fill” method was used to adjust for this bias by imputing potentially missing study results (Viechtbauer, 2010). Results remained qualitatively similar. These overall positive effects were not different for different latitudes or longitudes, for different annual precipitation at the study site, between natural and artificial tree holes or for insect‐only studies vs. studies with other organisms besides insects included (Table S2). However, we found a positive effect of annual mean temperature at the site on the strength of the relationship of tree‐hole size and organism richness (Figure 5c, Table S2), meaning that tree‐hole size increased organism richness more strongly under higher annual mean temperatures.

Most of the species in the tree holes depend directly or indirectly on detritus. Thus, many tree‐hole studies measured effects of detritus amount on communities (Figure 3). These studies mostly found positive effects of detritus amount on abundance and richness of organisms (Figure 4 and Figure S5). Our meta‐analysis also showed that the effects of detritus amount on abundance (df = 20, *Z* = 6.59, *p* < .001, Figure 5a) and richness (df = 17, Z = 3.81, *p* = .001, Figure 5b) were overall positive and significant. Funnel plots (Figure S6) and regression tests for asymmetry indicated publication bias in the effect of detritus amount on organism abundance. Results remained qualitatively similar when using the “trim‐and‐fill” method to adjust for this bias by imputing potentially missing study results (Viechtbauer, 2010). The positive effect of detritus amount on organism abundance was not different for different latitudes, for different annual mean temperature or annual precipitation at the site of the study or between insect‐only studies vs. studies with other organisms besides insects included (Table S2). However, the positive effect was stronger in natural than in artificial tree holes (Figure 5a, Table S2), and we found a weaker effect at higher longitudes (Table S2). The positive effect of detritus amount on organism richness was not different between artificial and natural tree holes or between insect‐only studies vs. studies with other organisms besides insects included (Table S2). However, we found stronger positive effects of detritus amount on organism richness at higher absolute latitude (Figure 5d, Table S2), weaker effects at higher longitude (Table S2), and stronger effects at higher annual mean temperature (Figure 5e, Table S2) and annual precipitation (Figure 5f, Table S2). This shows that detritus amount increased richness more strongly at higher latitudes, lower longitudes, higher annual mean temperature, and higher precipitation. Our additional analyses showed that the effects of detritus amount on abundance is still positive and significant when accounting for the effect of volume on detritus (df = 9, *Z* = 3.96, *p* < .001). Using the trim‐and‐fill method to account for asymmetry did not change this result. None of the moderators we tested had a significant influence on this effect. The effect of detritus on richness was no longer significant when we controlled for the effect of volume on detritus amount (df = 8, *Z* = 1.37, *p* = .171). There was a small significant difference between artificial and natural tree holes in this case with natural tree holes showing a slightly larger effect of detritus on richness than artificial ones. Detritus quality on the other hand was rarely studied (Figure 3). The few studies addressing this topic found that animal detritus as a sole resource clearly lead to higher abundance and richness of tree‐hole inhabiting organisms compared with plant detritus as a sole resource, but there were also differences among different species of plant detritus (Figure 4).

Many studies only sampled tree holes close to the ground (e.g., Paradise, 2004; Srivastava, 2005; Verdonschot et al., 2008), mostly for practical reasons. However, some observational studies measured and analyzed height from the ground as one environmental parameter (Figure 3a), and a few experiments even explicitly manipulated it (Figure 3b). Those studies found positive or no effects of height on organism abundance but negative or positive (or rarely, neutral) effects of height on richness (Figure 4).

The effects of physical and chemical tree‐hole water parameters on the inhabiting communities were also measured by many studies (Figure 3a) but less often manipulated in experiments (Figure 3b). Most commonly addressed were water temperature, dissolved oxygen content, pH and conductivity, and various effects of these variables on tree‐hole communities have indeed been shown (e.g., Blakely & Didham, 2010; Paradise, 1998; Schmidl et al., 2008; Yanoviak et al., 2006). For example, Yanoviak et al. (2006) found a positive effect of dissolved oxygen on insect richness in artificial tree holes and a negative effect in natural ones. Conductivity and pH had no effect on richness or abundance in this study. Water chemistry has also been shown to affect community composition (Gossner, Lade, et al., 2016). These effects may result from oviposition preferences of adults for certain environmental conditions (Paradise & Dunson, 1997; Schmidl et al., 2008). However, it is also possible that certain physical or chemical parameters determine larval survival rather than adult oviposition. The effect of varying tree‐hole density on tree‐hole communities was rarely measured (Figure 3a) and to our knowledge never explicitly manipulated (Figure 3b) despite its likely importance for the colonization of tree‐hole organisms with different dispersal ability. The few observational studies that addressed tree‐hole density found a negative or positive effect on abundance and an insignificant or a positive effect on richness (Figure 4). Furthermore, effects of natural or experimentally manipulated predation on tree‐hole communities were measured in a few studies (Figure 3). Results were inconsistent, showing negative effects on abundance but positive or negative effects on richness (Figure 4).

## 4 DISCUSSION

### 4.1 Organisms

Most studies we found recorded only few or even a single species and focused on mosquitoes or other disease‐transmitting insects. Outside of Europe and North America, few studies on tree‐hole communities (i.e., studies on more than a few selected species) have been conducted and those were often exclusively on insects. Tropical studies on other tree‐hole organisms such as microbes or mesofauna (e.g., nematodes and rotifers) would be strongly needed to assess their importance in these systems. However, they are often limited by the lack of experts on their taxonomy. Morphotype approaches are often used (see e.g., Gossner, Lade, et al., 2016) but render comparisons across studies difficult. Barcoding approaches are sometimes hampered by the few species records available in databases. Thus, there is a strong need for a reliable barcode reference database of organisms inhabiting tree holes.

Trait‐based analyses are now being used more and more in arthropods (e.g., Birkhofer et al., 2015; Wong et al., 2019). For tree holes, almost no trait‐based analyses of communities have been attempted so far (but see Petermann et al., 2020) but could lead to new insights into their metacommunity dynamics (e.g., dispersal‐related traits) and food webs (e.g., feeding traits) and allow for cross‐study comparisons even if identification to species level is not possible. Moreover, a trait‐based approach could help to analyze the consequences of climate change (e.g., increasing drought events) on community functional resistance and resilience, as trait‐based approaches in bromeliad‐inhabiting communities have shown (Céréghino et al., 2018; Marino et al., 2020; Srivastava et al., 2020; Srivastava et al., 2022).

### 4.2 Environmental effects

We found that there is a positive effect of tree‐hole size on abundance and richness of the organisms. This effect may reflect oviposition decisions (Bradshaw & Holzapfel, 1988) or mortality due to increased drought or lower resources in smaller holes (Gossner, Lade, et al., 2016). Our meta‐analysis showed a stronger effect of size on richness under high mean annual temperatures. This increased effect could be a signature of larger species pools in these areas but may also be a result of an increased drought risk (and thus, a bigger advantage of larger holes) in warmer sites. On the contrary, it may be advantageous to colonize small holes that may constitute habitats with lower competition (Fincke, 1992) or predation (Paradise et al., 2008), and this may be the reason that even very small tree holes contain organisms (Paradise, 2004). Therefore, the species that live in small tree holes might have specific traits, for example, they are adapted to drought (e.g., via resting stages), they are fast colonizers or they have short larval development times, but they are inferior competitors or sensitive to predation.

Similarly, detritus amount had a general positive effect on tree‐hole organism abundance and richness, but this might be a consequence of a correlation with tree‐holes size. The latter is supported by our finding that the effect of detritus amount on richness disappeared when we controlled for differences in tree‐hole volume in the subset of studies for which we had sufficient data. However, the generally strong and positive effect of detritus amount on abundance remained even when accounting for differences in size, indicating that resource amount per se exerts strong bottom‐up control on abundance. Detritus quality was also found to be important (e.g., Yee & Juliano, 2007). Most of the species in the tree holes live directly or indirectly on detritus as primary production may be restricted by low light levels (but see Ptatscheck & Traunspurger, 2015), but additional nutrients enter the system by stemflow water (Carpenter, 1982). Potentially, high precipitation decreases drought risk and may allow tree‐hole communities to exploit detrital resources better, explaining the stronger effect of detritus on richness that we found in our meta‐analysis at sites with high precipitation.

Our results show that abundance of tree‐hole organisms is often higher when tree holes are located higher up the tree compared with tree holes close to the ground (e.g., see Blakely & Didham, 2010; Gossner, Lade, et al., 2016). In contrast, species richness or diversity is sometimes lower when the tree hole is located higher up the tree, with additional differences in community composition (Blakely & Didham, 2010, Gossner, Lade, et al., 2016). The reasons may be different abiotic conditions in the canopy (smaller tree holes, higher frequency of drought, less resource input, and faster development due to faster warming of the water; Gossner, 2018) or differences in dispersal (Blakely & Didham, 2010, Gossner, Lade, et al., 2016).

We show that many studies measured physical or chemical parameters in the tree‐hole water. Temperature, dissolved oxygen content, pH, and conductivity were measured most often, and effects of these variables on tree‐hole communities have been shown (e.g., Blakely & Didham, 2010; Paradise, 1998; Yanoviak et al., 2006). More detailed water chemistry measurements (e.g., nitrate, ammonium, and phosphate) are taken more rarely (but see Schmidl et al., 2008; Gossner, Lade, et al., 2016; Petermann et al., 2016). Characteristics of the surrounding environment such as wind speed and direction, canopy cover, and relative humidity are also rarely measured (but see Khazan et al., 2015; Petermann et al., 2020) but may be important to describe, for example, conditions for dispersal. Unfortunately, community studies rarely test and compare responses of individual species to environmental parameters (but see Schmidl et al., 2008; Gossner & Petermann, 2022).Thus, whether tree‐hole species have environmental limits and/or habitat preferences is largely unknown.

We found few differences in effects of the environment on tree‐hole communities between natural and artificial tree holes in our meta‐analysis. Only the effect of detritus on abundance was stronger for natural tree holes. However, studies directly comparing the communities of natural and artificial tree holes found that communities in natural tree holes have a higher species richness but a lower density of organisms (Blakely et al., 2012; Petermann et al., 2016) and that community composition differs (Blakely et al., 2012; Petermann et al., 2016; Yanoviak et al., 2006), possibly also due to differences in abiotic parameters (e.g., higher temperature in artificial tree holes; Petermann et al., 2016). However, relatively few species seem to be exclusive to natural tree holes (Blakely et al., 2012; Petermann et al., 2016; Yanoviak et al., 2006). For those species, their absence in artificial tree holes may also be due to the often younger age of artificial tree holes (Yanoviak et al., 2006). Especially, species that have been successfully spreading into new areas are able to use artificial containers but may also use natural tree holes as habitats in their new range. One example of such habitat flexibility of novel species is the mosquito *Aedes albopictus* in the United States (Westby et al., 2020). Generally, differences in community composition between natural and artificial tree holes—with some exceptions—seem to arise from differences in (relative) abundances and less from differences in the presence and absence of species. Because environmental effects (such as the effect of detritus amount or forest management on species richness) can be detected in a similar way in artificial and natural tree holes, many studies (e.g., Blakely et al., 2012; Petermann et al., 2016) suggest—and we agree—that artificial tree holes can be useful analogs of natural tree holes.

### 4.3 Spatial dynamics and metacommunity aspects

We show that some of the few studies addressing tree‐hole density indeed demonstrate effects on inhabitant abundance and sometimes richness (e.g., Gossner, Lade, et al., 2016; Petermann et al., 2020) suggesting that dispersal limitation exists. In contrast, some studies (e.g., Schulz et al., 2012) found no effect of space on passively or actively dispersing invertebrates and habitat properties might be more important than spatial distance in structuring the communities at small spatial scales (Gossner, unpubl. results). Adult insects are active dispersers with sometimes strong dispersal abilities, but which still differ between species (Sarremejane et al., 2020) and are often unknown, except for certain large, charismatic species such as damselflies for which population genetics and dispersal experiments allow estimations of dispersal distances (Fincke, 2006). Other species, such as bacteria and protists, are passive dispersers depending on biotic (insects, birds) or abiotic vectors (wind) which may make their dispersal more erratic but nevertheless may lead to relatively high dispersal distances. However, Bell (2010) found that bacterial communities in tree holes were spatially structured, at least over short time scales, indicating dispersal limitation. Few studies have made an attempt to sample or at least map all tree holes in the sampling area (but see Kitching, 1969; Gossner, Lade, et al., 2016; Petermann et al., 2016; Petermann et al., 2020) or other water sources such as ponds. This mapping would be required to assess potential sources of colonists. Water‐filled tree holes are a textbook example of metacommunities. Still, hardly any studies have explicitly addressed aspects of metacommunity theory in tree holes (but see Ellis et al., 2006).

### 4.4 Trophic interactions and ecosystem functions within tree holes and at the aquatic–terrestrial interface

Our results show that relatively few observational and experimental studies considered top‐down processes, but these indeed found negative effects of predation on abundance, and negative or positive effects on richness, for example, a strong effect of a top predator mosquito species (*Toxorhynchites*) on other dipteran larvae (Paradise et al., 2008; Smith et al., 2009). These examples are from the tropics where predators occur in the tree‐hole food web and their top‐down effects can be tested. Intriguingly, in Central European, tree holes obligate predators of insects seem to be absent or very rare (Kitching, 1971; Rohnert, 1950). Some species are suspected to be facultative predators, but feeding trials or stable isotope techniques (e.g., reviewed for arthropods by Hood‐Nowotny & Knols, 2007, for a soil food web example see Digel et al., 2014) would be necessary to unequivocally confirm this notion. External terrestrial predators such as birds, carabids, or spiders could also make use of aquatic species as a resource, especially under drought conditions (Srivastava, 2005), but this cross‐system predation has rarely been studied (but see Gossner et al., 2020; Kirsch et al., 2021).

Decomposition is the most important ecosystem function in the tree‐hole system, and the inhabiting insect species are mostly detritivores of several distinct functional groups: scrapers (e.g., scirtid beetle larvae), shredders (e.g., tipulid larvae), collector‐gatherers (e.g., many chironomid larvae), and filter feeders (e.g., many mosquito larvae). Processing chains between these insect larvae have been shown for tree holes (Paradise, 1999). Aquatic fungi (Sridhar et al., 2013), bacteria (Verdonschot et al., 2008), protists (Walker et al., 2010), mesofauna (Ptatscheck & Traunspurger, 2015), astigmatic mites (Fashing, 1998), annelids (e.g., genus *Aelosoma*), and protists can be important decomposers but have rarely been studied in terms of their contribution to the processing chains of water‐filled tree holes.

While detritus amount is one of the most commonly measured variables of tree‐hole studies, it is surprising that the actual process of decomposition is rarely investigated. Kaufman et al. (2008) did not find an effect of insect larva presence on mass loss of leaves in tree holes. Verdonschot et al. (2008) assessed nutrient fluxes and microbial processes in a very detailed study in a single tree hole over 4 months through different periods of rainfall and drought. They found that tree holes may exist in different states: isolated during dry weather and “connected” during rain events, when stem flow and throughfall provide variable nutrient pulses. Such short‐distance connection between adjacent systems via overflowing water has also been shown to occur in rock pools (Sciullo & Kolasa, 2012).

Productivity of the tree‐hole system could be measured at several trophic levels, for example, as microbial productivity increasing invertebrate abundance and richness (Yee et al., 2007; Yee & Juliano, 2007). Meso‐ and macrofaunal components of temperate tree holes showed lower secondary production per square meter when compared with a temperate lake ecosystem (Ptatscheck & Traunspurger, 2015). However, tropical bromeliad phytotelmata can reach much higher values of secondary production than larger tropical aquatic systems (Dézerald et al., 2018), and it would be very interesting to measure and compare secondary production in tropical tree holes to these aquatic systems. At the insect level, productivity could also be measured as insect biomass leaving the tree hole, acting as a reverse subsidy to the terrestrial ecosystem (Dreyer et al., 2015) but also as a potential source of disease vectors. Studies on mosquito emergence rates showed that they were unrelated to detritus input (Walker & Merritt, 1988), but that higher emergence rates can be expected from larger holes (Washburn et al., 1989), especially in urban areas where water temperatures are higher (Li et al., 2014).

### 4.5 Seasonal and temporal dynamics

Input into the tree‐hole system in temperate regions is dominated by the strongly seasonal process of leaf fall. In tropical systems, leaf fall can still be partly synchronized between and within tree species (Lieberman & Lieberman, 1984). The major output from the system, insect emergence, follows the seasonal life cycles of the insects. Temporal differences in insect abundance, richness, and composition have been found for tropical tree holes (Sanchez & Liria, 2009; Yanoviak et al., 2006) and temperate ones (Devetter, 2004; Harlan & Paradise, 2006; Paradise et al., 2008). However, tree hole communities have been shown to be structured by drought, rainfall events, or freezing conditions, rather than strict seasonality (Gossner, 2018). Abiotic variables such as pH, dissolved oxygen, and conductivity also fluctuate temporally (Harlan & Paradise, 2006; Sanchez & Liria, 2009; Yanoviak et al., 2006) as well as microbial communities (Verdonschot et al., 2008), and their variability may explain changes in insect community composition. Interesting but little explored aspects of dynamics in tree holes would be the change in biological interactions and processes over time (e.g., shown by Kitching, 1987b; Smith et al., 2009). Intriguingly, not much is known about the fate of temperate tree‐hole systems in winter. Tree‐hole organisms generally differ in their overwintering stages and strategies, for example, mosquitoes overwinter as eggs, whereas scirtid beetles overwinter as larvae (Barrera, 1996). Tree holes may actually freeze down to the bottom regularly (Gossner, 2018). Most species overwintering in larval stage likely find a way to shelter from the cold in crevices or have other survival strategies but mortality rates may be high during long periods of frost (Gossner, 2018; Rohnert, 1950). A similarly strong disturbance to tree‐hole communities is drought, which, depending on hole size, shape, location, and climate, may occur several times a year and may strongly alter communities since species differ in their sensitivity to drought (Srivastava, 2005). Again, organisms may survive in crevices or have desiccation‐resistant eggs or dormant larvae stages (Benoit, 2010; Juliano et al., 2002) and quickly re‐emerge after rain (Gossner, 2018; Srivastava, 2005). The initial colonization or recolonization of habitats after drought is not very well studied but can be very fast, with nematodes and tardigrades arriving within 1 week, and insect larvae appearing after about 8 weeks (Ptatscheck & Traunspurger, 2014) or much faster for mosquito larvae (own observation). However, for full communities to develop, much more time may be necessary since some species are much slower to arrive and have longer life cycles (Yanoviak, 2001a).

### 4.6 Anthropogenic influence

The few studies on the effect of anthropogenic environmental change on tree‐hole communities suggest that there are strong negative effects of land‐use change (Khazan et al., 2015; Yanoviak et al., 2006), forest fragmentation (Nicholas, 2016), and forest management intensity (Gossner, Lade, et al., 2016; Petermann et al., 2020, 2016), largely operating in an indirect way by affecting habitat and resource availability of the species (see e.g., Gossner, Lade, et al., 2016; Petermann et al., 2020).

Tree holes in urban areas are so far exclusively studied with respect to mosquito species, mainly in the tropics (e.g., Mangudo et al., 2015; Weterings et al., 2014). There are only a low number of studies from temperate systems (Chaves et al., 2011; Leisnham et al., 2006; Schaffner et al., 2009) because mosquitoes do not pose a large problem as vectors of diseases there yet. However, with a changing climate and strongly increased global transport, some of these mosquito species and some of the diseases they carry have been shown to move northward, for example in Europe, invade new locations (Cunze et al., 2016; Medlock et al., 2012; Thomas et al., 2018), and might cause problems to native species and forest health (Suter et al., 2017). Tree‐hole organisms such as bacteria may also be strongly affected by environmental pollution, for example, with pesticides (Ager et al., 2010). Thus, natural or artificial tree holes may indeed constitute indicators of environmental change and anthropogenic impacts (Petermann et al., 2016) since they are small systems, easy to investigate, and very sensitive to environmental change in their surroundings.

### 4.7 Avenues for future research

Whereas quite some data exists on organisms living in water‐filled tree holes, there are still large knowledge gaps regarding these systems (Table 1), specifically on how their food webs function, how they interact with other aquatic and the surrounding terrestrial ecosystems, and what roles potential aquatic or terrestrial predators play. More advanced techniques such as stable isotope or gut content analyses with barcoding approaches but also simple methods such as feeding trials, body‐size, or other straightforward trait measurements (incl. Intraspecific ones) or the use of artificial larvae to measure terrestrial predator attack rates are valuable in this context. In particular, the interaction between aquatic and terrestrial ecosystems could be measured based on biomass fluxes (e.g., emergence rates) or long‐chain polyunsaturated fatty acids (PUFAs, Hixson et al., 2015) and would help to describe the ecosystem functions and energy flows (Buzhdygan et al., 2020) in meta‐ecosystem approaches.

**TABLE 1:** Overview over important gaps in tree‐hole research that we identified in this study and suggestions for future research that could contribute to filling these gaps. For details see text

Identified research gaps	Suggestions for future research
Food webs in tree holes and interactions with other aquatic and terrestrial systems	Stable isotope analyses of food webs Gut content analyses and barcoding approaches Feeding trials Body‐size or other trait measurements Use of artificial larvae to measure predator attack rates Biomass‐based measurements of trophic levels and processes to describe energy flows of the meta‐ecosystem Fatty‐acid based analyses of nutritious quality (PUFAs)
Temporal dynamics (effects of drought, overflow, freezing and other seasonal and non‐seasonal dynamics)	Sequential harvesting of replicate systems (space for time substitution) eDNA approaches to follow the organisms in tree holes over time without destroying the systems RNA‐based approaches, measuring only the active organisms
Using natural gradients to test effects of environmental parameters on communities	Studies along altitudinal and latitudinal gradients Large‐scale observational studies using the same methodology Global experiments
Using tree holes as model systems to study biodiversity‐ecosystem functioning relationships, community assembly mechanisms and metacommunity dynamics	Manipulation of horizontal and vertical diversity Manipulation of multiple stressors (e.g., climate‐change related, such as temperature and precipitation) Manipulation of spatial arrangement and properties of tree holes Studying dispersal of individual species
Using tree holes as indicator systems of the effects of environmental change on ecosystems	Further test effects of various environmental changes (e.g., land use and urbanization) on natural and artificial tree‐hole communities Test relationship of natural tree‐hole presence with species diversity of various organism groups

Temporal dynamics of tree holes such as changes in water regimes including drought and conditions of high stemflow, freezing, and other seasonal and nonseasonal dynamics are likely among the most important drivers of their community structure. By investigating these effects, tree‐hole research could help to understand effects of rapid environmental change on many ecosystems. One difficulty of sequential sampling to assess temporal dynamics in communities is that the system will be destroyed or at least strongly disturbed in the process. Another option is sequential harvesting of replicate systems in a “space for time substitution” (Jenkins et al., 1992; Petermann et al., 2016; Ptatscheck & Traunspurger, 2015), but the results may be misleading due to differences between individual systems. Potentially, eDNA (Carraro et al., 2020; Pawlowski et al., 2018; Pawlowski et al., 2020) could be used to follow the organisms over time without destroying the systems. However, we still lack knowledge on DNA persistence in the tree‐hole system and contamination by stemflow water or animal feces. Therefore, RNA‐based approaches might be more promising by measuring only the active organisms, yet not well established, and very cost‐intensive (Brandt et al., 2020). More research is needed on the most reliable standardized method for tracking temporal community dynamics in tree‐hole systems.

Tree‐hole studies so far make only limited use of natural environmental gradients to test effects of environmental parameters on communities. Comparisons between tropical and temperate tree holes have rarely been made (but see Srivastava, 2005), and only one study used an explicit latitudinal gradient, showing that richness of species inhabiting each tree hole increased with latitude (Yee et al., 2007). Large‐scale or even global observational or experimental tree‐hole studies using the same methodology could be very useful in this respect.

Natural and artificial tree holes can serve as model systems for studying fundamental ecological questions, for example, productivity–richness relationships (Srivastava & Lawton, 1998), local–global richness patterns (Srivastava, 2005), and metacommunity concepts (Ellis et al., 2006). In addition, multiple stressors (e.g., climate‐change related, such as temperature and precipitation) as well as horizontal (within trophic guild) and vertical diversity (across trophic guilds) of the communities can be manipulated, and this would allow for testing the resistance and resilience of biodiversity–ecosystem functioning relationships. Furthermore, community assembly mechanisms and metacommunity dynamics could be addressed by experimentally manipulating the spatial arrangement and properties of these microhabitats and by studying species dispersal, for example, by population genetic studies or isotope labeling.

From an applied perspective, tree holes could, after filling some of the described knowledge gaps, be used as indicator systems of environmental change(Ager et al., 2010; Petermann et al., 2016), especially also in urban areas. Likewise, the presence of tree holes themselves can been used as a tool to assess the habitat quality of forests (Asbeck et al., 2021; Kraus et al., 2016; Larrieu et al., 2018).

## 5 CONCLUSIONS

In summary, we found that the research on water‐filled tree holes conducted in the last 100 years has shown strong and positive effects of tree‐hole size and detritus amount on organism abundance and richness, with modulating effects of temperature, longitude, latiude, and the type of tree hole (natural vs. artificial tree holes). We also showed that parameters such as height of the tree hole above ground, tree‐hole density, predation, and detritus type can be important drivers of community structure but are less often tested. Future studies should investigate the structure, functions, and temporal dynamics of tree‐hole food webs and their cross‐system interactions. In addition, global observational or experimental tree‐hole studies are needed to collect data on the natural spatial variation of communities and environmental effects. The information gathered on tree holes in the past together with the data we will collect in the future will hopefully lead to an improved understanding of these unique aquatic islands in terrestrial ecosystems and their use as model and indicator systems to track responses of communities and ecosystem functions in response to environmental change.

## AUTHOR CONTRIBUTIONS

**Jana S. Petermann:** Conceptualization (lead); formal analysis (lead); methodology (lead); writing – original draft (lead); writing – review and editing (lead). **Martin M. Gossner:** Conceptualization (equal); writing – review and editing (equal).

## CONFLICT OF INTEREST

The authors declare no competing interests.

## Supporting information


Figure S1

Figure S2

Figure S3

Figure S4

Figure S5

Figure S6

Table S1‐S2

## Data Availability

Data used for the meta‐analyses is available from Dryad, https://doi.org/10.5061/dryad.08kprr55f .

## References

[ece39206-bib-0001] Ager, D. , Evans, S. , Li, H. , Lilley, A. K. , & Van Der Gast, C. J. (2010). Anthropogenic disturbance affects the structure of bacterial communities. Environmental Microbiology, 12, 670–678. 20002134 10.1111/j.1462-2920.2009.02107.x

[ece39206-bib-0002] Asbeck, T. , Großmann, J. , Paillet, Y. , Winiger, N. , & Bauhus, J. (2021). The use of tree‐related microhabitats as Forest biodiversity indicators and to guide integrated Forest management. Current Forestry Reports, 7, 59–68.

[ece39206-bib-0003] Barrera, R. (1996). Species concurrence and the structure of a community of aquatic insects in tree holes. Journal of Vector Ecology, 21, 66–80.

[ece39206-bib-0004] Bell, T. (2010). Experimental tests of the bacterial distance‐decay relationship. ISME Journal, 4, 1357–1365. 20535220 10.1038/ismej.2010.77

[ece39206-bib-0005] Bell, T. , Ager, D. , Song, J.‐I. , Newman, J. A. , Thompson, I. P. , Lilley, A. K. , & van der Gast, C. J. (2005). Larger Islands house more bacterial taxa. Science, 308, 1884. 15976296 10.1126/science.1111318

[ece39206-bib-0006] Benoit, J. B. (2010). Water management by dormant insects: Comparisons between dehydration resistance during summer aestivation and Winter diapause. In C. Arturo Navas & J. E. Carvalho (Eds.), Aestivation: Molecular and physiological aspects (pp. 209–229). Springer. 10.1007/978-3-642-02421-4_10 20069411

[ece39206-bib-0007] Birkhofer, K. , Smith, H. G. , Weisser, W. W. , Wolters, V. , & Gossner, M. M. (2015). Land‐use effects on the functional distinctness of arthropod communities. Ecography, 38, 889–900.

[ece39206-bib-0008] Blakely, T. J. , & Didham, R. K. (2010). Disentangling the mechanistic drivers of ecosystem‐size effects on species diversity. Journal of Animal Ecology, 79, 1204–1214. 20636346 10.1111/j.1365-2656.2010.01729.x

[ece39206-bib-0009] Blakely, T. J. , Harding, J. S. , & Didham, R. K. (2012). Distinctive aquatic assemblages in water‐filled tree holes: A novel component of freshwater biodiversity in New Zealand temperate rainforests. Insect Conservation and Diversity, 5, 202–212.

[ece39206-bib-0010] Bradshaw, W. E. , & Holzapfel, C. M. (1988). Drought and the organization of tree‐hole mosquito communities. Oecologia, 74, 507–514. 28311756 10.1007/BF00380047

[ece39206-bib-0011] Brandt, M. I. , Trouche, B. , Henry, N. , Liautard‐Haag, C. , Maignien, L. , de Vargas, C. , Wincker, P. , Poulain, J. , Zeppilli, D. , & Arnaud‐Haond, S. (2020). An assessment of environmental metabarcoding protocols aiming at favoring contemporary biodiversity in inventories of Deep‐Sea communities. Frontiers in Marine Science, 7, 234.

[ece39206-bib-0012] Brehm, V. (1925). Hängende Aquarien in der Pflanzenwelt. Mikrokosmos, 19, 1–6.

[ece39206-bib-0013] Buzhdygan, O. Y. , Meyer, S. T. , Weisser, W. W. , Eisenhauer, N. , Ebeling, A. , Borrett, S. R. , Buchmann, N. , Cortois, R. , De Deyn, G. B. , de Kroon, H. , Gleixner, G. , Hertzog, L. R. , Hines, J. , Lange, M. , Mommer, L. , Ravenek, J. M. , Scherber, C. , Scherer‐Lorenzen, M. , Scheu, S. , … Petermann, J. S. (2020). Biodiversity increases multitrophic energy‐use efficiency, flow and storage in grasslands. Nature Ecology and Evolution, 4, 393–405. 32094542 10.1038/s41559-020-1123-8

[ece39206-bib-0014] Carlson, J. , Keating, J. , Mbogo, C. M. , Kahindi, S. , & Beier, J. C. (2004). Ecological limitations on aquatic mosquito predator colonization in the urban environment. Journal of Vector Ecology: Journal of the Society for Vector Ecology, 29, 331–339. 15707292 PMC3705640

[ece39206-bib-0015] Carpenter, S. R. (1982). Stemflow chemistry: Effects on population dynamics of detritivorous mosquitoes in tree‐hole ecosystems. Oecologia, 53, 1–6. 28310595 10.1007/BF00377128

[ece39206-bib-0016] Carraro, L. , Mächler, E. , Wüthrich, R. , & Altermatt, F. (2020). Environmental DNA allows upscaling spatial patterns of biodiversity in freshwater ecosystems. Nature Communications, 11, 3585. 10.1038/s41467-020-17337-8 PMC7367889 32680984

[ece39206-bib-0017] Cebrián‐Camisón, S. , Martínez‐de la Puente, J. , & Figuerola, J. (2020). A literature review of host feeding patterns of invasive Aedes mosquitoes in Europe. Insects, 11, 848. 10.3390/insects11120848 PMC7760726 33260438

[ece39206-bib-0018] Céréghino, R. , Pillar, V. D. , Srivastava, D. S. , de Omena, P. M. , MacDonald, A. A. M. , Barberis, I. M. , Corbara, B. , Guzman, L. M. , Leroy, C. , Ospina Bautista, F. , Romero, G. Q. , Trzcinski, M. K. , Kratina, P. , Debastiani, V. J. , Gonçalves, A. Z. , Marino, N. A. C. , Farjalla, V. F. , Richardson, B. A. , Richardson, M. J. , … Montero, G. (2018). Constraints on the functional trait space of aquatic invertebrates in bromeliads. Functional Ecology, 32, 2435–2447.

[ece39206-bib-0019] Chaves, L. F. , Hamer, G. L. , Walker, E. D. , Brown, W. M. , Ruiz, M. O. , & Kitron, U. D. (2011). Climatic variability and landscape heterogeneity impact urban mosquito diversity and vector abundance and infection. Ecosphere, 2, 1–21.

[ece39206-bib-0020] Christophers, S. R. , & Chand, K. (1916). A tree‐hole breeding *anopheles* from southern India. *A*. (*Coelodiarzesis*) *culiciformi*s, Cogill. Indian Journal of Medical Research Calcutta, 3, 638–645.

[ece39206-bib-0021] Cumberlidge, N. , Fenolio, D. B. , Walvoord, M. E. , & Stout, J. (2005). Tree‐climbing crabs (Potamonautidae and Sesarmidae) from Phytotelmic microhabitats in rainforest canopy in Madagascar. Journal of Crustacean Biology, 25, 302–308.

[ece39206-bib-0022] Cunze, S. , Koch, L. K. , Kochmann, J. , & Klimpel, S. (2016). *Aedes albopictus* and *Aedes japonicus* ‐ two invasive mosquito species with different temperature niches in Europe. Parasites & Vectors, 9, 573. 27814747 10.1186/s13071-016-1853-2 PMC5097377

[ece39206-bib-0023] Devetter, M. (2004). Invertebrate fauna of treeholes in relation to some habitat conditions in southern Bohemia (Czech Republic). Acta Societatis Zoologicae Bohemicae, 68, 161–168.

[ece39206-bib-0024] Dézerald, O. , Leroy, C. , Corbara, B. , Dejean, A. , Talaga, S. , & Céréghino, R. (2018). Tank bromeliads sustain high secondary production in neotropical forests. Aquatic Sciences, 80, 14.

[ece39206-bib-0025] Digel, C. , Curtsdotter, A. , Riede, J. , Klarner, B. , & Brose, U. (2014). Unravelling the complex structure of forest soil food webs: Higher omnivory and more trophic levels. Oikos, 123, 1157–1172.

[ece39206-bib-0026] Dreyer, J. , Townsend, P. A. , Iii, J. C. H. , Hoekman, D. , Vander Zanden, M. J. , & Gratton, C. (2015). Quantifying aquatic insect deposition from lake to land. Ecology, 96, 499–509. 26240871 10.1890/14-0704.1

[ece39206-bib-0027] Ellis, A. M. , Lounibos, L. P. , & Holyoak, M. (2006). Evaluating the long‐term metacommunity dynamics of treehole mosquitoes. Ecology, 87, 2582–2590. 17089666 10.1890/0012-9658(2006)87[2582:etlmdo]2.0.co;2 PMC1828635

[ece39206-bib-0028] Erwin, T. L. (1983). Tropical forest canopies: The last biotic frontier. Bulletin of the Entomological Society of America, 29, 14–19.

[ece39206-bib-0029] Fashing, N. (1998). Functional morphology as an aid in determining trophic behaviour: The placement of astigmatic mites in food webs of water‐filled tree‐hole communities. Enperimental and Applied Acarology, 22, 435–453.

[ece39206-bib-0030] Fincke, O. (1992). Interspecific competition for tree holes: Consequences for mating systems and coexistence in neotropical damselflies. The American Naturalist, 139, 80–101.

[ece39206-bib-0031] Fincke, O. (2006). Use of forest and tree species, and dispersal by giant damselflies (Pseudostigmatidae): Their prospects in fragmented forests (pp. 103–125). Forests and Dragonflies. Sofia, Pensoft Publishers.

[ece39206-bib-0032] Fincke, O. M. , Yanoviak, S. P. , & Hanschu, R. D. (1997). Predation by odonates depresses mosquito abundance in water‐filled tree holes in Panama. Oecologia, 112, 244–253. 28307577 10.1007/s004420050307

[ece39206-bib-0033] Gönczöl, J. , & Révay, Á. (2003). Treehole fungal communities: Aquatic, aero‐aquatic and dematiaceous hyphomycetes. Fungal Diversity, 12, 19–34.

[ece39206-bib-0034] Gönczöl, J. , & Révay, A. (2004). Fungal spores in rainwater: Stemflow, throughfall and gutter conidial assemblages. Fungal Diversity, 16, 67–86.

[ece39206-bib-0035] Gossner, M. (2018). A three year study of the phenology of insect larvae (coleoptera, Diptera) in water‐filled tree holes in the canopy of a beech tree. European Journal of Entomology, 115, 524–534.

[ece39206-bib-0036] Gossner, M. , Lade, P. , Schober, A. , Sichardt, N. , Kahl, T. , Bauhus, J. , Weisser, W. W. , & Petermann, J. S. (2016). Effects of management on aquatic tree‐hole communities in temperate forests are mediated by detritus amount and water chemistry. Journal of Animal Ecology, 96, 428–439. 10.1111/1365-2656.12437 26332767

[ece39206-bib-0037] Gossner, M. M. , Gazzea, E. , Diedus, V. , Jonker, M. , & Yaremchuk, M. (2020). Using sentinel prey to assess predation pressure from terrestrial predators in water‐filled tree holes. European Journal of Entomology, 117, 226–234.

[ece39206-bib-0038] Gossner, M. M. , Lewinsohn, T. M. , Kahl, T. , Grassein, F. , Boch, S. , Prati, D. , Birkhofer, K. , Renner, S. C. , Sikorski, J. , Wubet, T. , Arndt, H. , Baumgartner, V. , Blaser, S. , Blüthgen, N. , Börschig, C. , Buscot, F. , Diekötter, T. , Jorge, L. R. , Jung, K. , … Allan, E. (2016). Land‐use intensification causes multitrophic homogenization of grassland communities. Nature, 540, 266–269. 27919075 10.1038/nature20575

[ece39206-bib-0039] Gossner, M. M. , & Petermann, J. S. (2022). Vertical stratification of insect species developing in water‐filled tree holes. Frontiers in Forests and Global Change, 4. 10.3389/ffgc.2021.816570

[ece39206-bib-0040] Greeney, H. F. (2001). The insects of plant‐held waters: A review and bibliography. Journal of Tropical Ecology, 17, 241–260.

[ece39206-bib-0041] Grinang, J. , Yong Min, P. , & Ng, P. K. L. (2015). A new species of tree‐hole dwelling freshwater crab of the genus Arachnothelphusa Ng, 1991 (crustacea: Decapoda: Brachyura: Gecarcinucidae) from northern Sarawak, Malaysia, Borneo. The Raffles Bulletin of Zoology, 63, 454–460.

[ece39206-bib-0042] Griswold, M. W. , & Lounibos, L. P. (2006). Predator identity and additive effects in a treehole community. Ecology, 87, 987–995. 16676542 10.1890/0012-9658(2006)87[987:piaaei]2.0.co;2 PMC1820834

[ece39206-bib-0043] Harlan, N. P. , & Paradise, C. J. (2006). Do habitat size and shape modify abiotic factors and communities in artificial treeholes? Community Ecology, 7, 211–222.

[ece39206-bib-0044] Hixson, S. M. , Sharma, B. , Kainz, M. J. , Wacker, A. , & Arts, M. T. (2015). Production, distribution, and abundance of long‐chain omega‐3 polyunsaturated fatty acids: A fundamental dichotomy between freshwater and terrestrial ecosystems. Environmental Reviews, 23, 414–424.

[ece39206-bib-0045] Hood‐Nowotny, R. , & Knols, B. G. J. (2007). Stable isotope methods in biological and ecological studies of arthropods. Entomologia Experimentalis et Applicata, 124, 3–16.

[ece39206-bib-0046] Hoshi, T. , Imanishi, N. , Higa, Y. , & Chaves, L. F. (2014). Mosquito biodiversity patterns around urban environments in south‐Central Okinawa Island, Japan. Journal of the American Mosquito Control Association, 30, 260–267. 25843131 10.2987/14-6432R.1

[ece39206-bib-0047] Inger, R. F. (1966). The systematics and zoogeography of the amphibia of Borneo. Field Museum of Natural History.

[ece39206-bib-0048] Jenkins, B. , Kitching, R. L. , & Pimm, S. L. (1992). Productivity, disturbance and food web structure at a local spatial scale in experimental container habitats. Oikos, 65, 249–255.

[ece39206-bib-0049] Juliano, S. A. , O'Meara, G. F. , Morrill, J. R. , & Cutwa, M. M. (2002). Desiccation and thermal tolerance of eggs and the coexistence of competing mosquitoes. Oecologia, 130, 458–469. 20871747 10.1007/s004420100811 PMC2944657

[ece39206-bib-0050] Kaufman, M. G. , Bland, S. N. , Worthen, M. E. , Walker, E. D. , & Klug, M. J. (2001). Bacterial and Fungal Biomass Responses to Feeding by LarvalAedes triseriatus (Diptera: Culicidae). Journal of Medical Entomology, 38(5), 711–719. 10.1603/0022-2585-38.5.711 11580044

[ece39206-bib-0051] Kaufman, M. G. , Chen, S. , & Walker, E. D. (2008). Leaf‐associated bacterial and fungal taxa shifts in response to larvae of the tree hole mosquito, Ochlerotatus triseriatus. Microbial Ecology, 55, 673–684. 17899246 10.1007/s00248-007-9310-6 PMC4053173

[ece39206-bib-0052] Khazan, E. S. , Bright, E. G. , & Beyer, J. E. (2015). Land management impacts on tree hole invertebrate communities in a neotropical rainforest. Journal of Insect Conservation, 19, 681–690.

[ece39206-bib-0053] Kirsch, J.‐J. , Sermon, J. , Jonker, M. , Asbeck, T. , Gossner, M. M. , Petermann, J. S. , & Basile, M. (2021). The use of water‐filled tree holes by vertebrates in temperate forests. Wildlife Biology, 2021(1), 1–4.

[ece39206-bib-0054] Kitching, R. L. (1969). The fauna of tree‐holes in relation to environmental factors. PhD thesis. University of Oxford.

[ece39206-bib-0055] Kitching, R. L. (1971). An ecological study of water‐filled tree‐holes and their position in the woodland ecosystem. Journal of Animal Ecology, 40, 281–302.

[ece39206-bib-0056] Kitching, R. L. (1987a). A preliminary account of the metazoan food webs in phytotelmata from Sulawesi, Indonesia. Malayan Nature Journal, 41, 1–12.

[ece39206-bib-0057] Kitching, R. L. (1987b). Spatial and temporal variation in food webs in water‐filled treeholes. Oikos, 48, 280–288.

[ece39206-bib-0058] Kitching, R. L. (2000). Food webs and container habitats. Cambridge University Press.

[ece39206-bib-0059] Kitching, R. L. (2001). Food webs in phytotelmata: "bottom‐up" and "top‐down" explanations for community structure. Annual Review of Entomology, 46, 729–760. 10.1146/annurev.ento.46.1.729 11112185

[ece39206-bib-0060] Kraus, D. , R. Bütler , F. Krumm , T. Lachat , L. Larrieu , U. Mergner , Y. Paillet , T. Rydkvist , A. Schuck , and S. Winter . 2016. Catalogue of tree microhabitats ‐ reference field list integrate+ technical paper.

[ece39206-bib-0061] Lardner, B. , & bin Lakim, M. (2002). Tree‐hole frogs exploit resonance effects. Nature, 420, 475. 12466831 10.1038/420475a

[ece39206-bib-0062] Larrieu, L. , Paillet, Y. , Winter, S. , Bütler, R. , Kraus, D. , Krumm, F. , Lachat, T. , Michel, A. K. , Regnery, B. , & Vandekerkhove, K. (2018). Tree related microhabitats in temperate and Mediterranean European forests: A hierarchical typology for inventory standardization. Ecological Indicators, 84, 194–207.

[ece39206-bib-0063] Leibold, M. A. , Holyoak, M. , Mouquet, N. , Amarasekare, P. , Chase, J. M. , Hoopes, M. F. , Holt, R. D. , Shurin, J. B. , Law, R. , Tilman, D. , Loreau, M. , & Gonzalez, A. (2004). The metacommunity concept: A framework for multi‐scale community ecology. Ecology Letters, 7, 601–613.

[ece39206-bib-0064] Leisnham, P. T. , Lester, P. J. , Slaney, D. P. , & Weinstein, P. (2006). Relationships between mosquito densities in artificial container habitats, land use and temperature in the Kapiti‐Horowhenua region, New Zealand. New Zealand Journal of Marine and Freshwater Research, 40, 285–297.

[ece39206-bib-0065] Li, Y. , Kamara, F. , Zhou, G. , Puthiyakunnon, S. , Li, C. , Liu, Y. , Zhou, Y. , Yao, L. , Yan, G. , & Chen, X.‐G. (2014). Urbanization increases Aedes albopictus larval habitats and accelerates mosquito development and survivorship. PLoS Neglected Tropical Diseases, 8, e3301. 25393814 10.1371/journal.pntd.0003301 PMC4230920

[ece39206-bib-0066] Lieberman, D. , & Lieberman, M. (1984). The causes and consequences of synchronous Flushing in a dry tropical Forest. Biotropica, 16, 193–201.

[ece39206-bib-0067] Maguire, B. J. (1971). Phytotelmata: Biota and community structure determination in plant‐held waters. Annual Review of Ecology and Systematics, 2, 439–464.

[ece39206-bib-0068] Magyar, D. , Vass, M. , & Oros, G. (2017). Dendrotelmata (water‐filled tree holes) as fungal hotspots ‐ a long term study. Cryptogamie, Mycologie, 38, 55–66.

[ece39206-bib-0069] Mangudo, C. , Aparicio, J. P. , & Gleiser, R. M. (2014). Notes on the occurrence and habitats of *Sabethes purpureus* in Salta Province, Argentina. Journal of the American Mosquito Control Association, 30, 57–60. 24772679 10.2987/13-6380.1

[ece39206-bib-0070] Mangudo, C. , Aparicio, J. P. , & Gleiser, R. M. (2015). Tree holes as larval habitats for Aedes aegypti in urban, suburban and forest habitats in a dengue affected area. Bulletin of Entomological Research, 105, 679–684. 26193903 10.1017/S0007485315000590

[ece39206-bib-0071] Marino, N. , Céréghino, R. , Gilbert, B. , Petermann, J. , Srivastava, D. , de Omena, P. , Bautista, F. , Guzman, L. , Romero, G. , Trzcinski, M. , Barberis, I. , Corbara, B. , Debastiani, V. , Dézerald, O. , Kratina, P. , Leroy, C. , MacDonald, A. A. , Montero, G. , Pillar, V. , … Farjalla, V. (2020). Species niches, not traits, determine abundance and occupancy patterns: A multi‐site synthesis. Global Ecology and Biogeography, 29, 295–308.

[ece39206-bib-0072] Medlock, J. M. , Hansford, K. M. , Schaffner, F. , Versteirt, V. , Hendrickx, G. , Zeller, H. , & Bortel, W. V. (2012). A review of the invasive mosquitoes in Europe: Ecology, public health risks, and control options. Vector Borne and Zoonotic Diseases, 12, 435–447. 22448724 10.1089/vbz.2011.0814 PMC3366101

[ece39206-bib-0073] Müller, F. , & Müller, H. (1878). Phryganiden‐Studien. Kosmos, 4, 386–396.

[ece39206-bib-0074] Nicholas, A. (2016). Forest fragmentation changes macroinvertebrate community composition in tropical tree holes. University of British Columbia.

[ece39206-bib-0075] Nishadh, K. A. , & Das, K. S. (2014). Tree‐hole aquatic habitats: Inhabitants, processes and experiments. A review. International Journal of Conservation Science, 5, 253–268.

[ece39206-bib-0076] Nöllert, A. (2012). Ungewöhnlicher Laichplatz des Grasfrosches, *Rana t. temporaria*, in einer Phytotelme im Nationalpark Hainich, Thüringen. Terraria, 38, 96–101.

[ece39206-bib-0077] O'Meara, G. F. , Gettman, A. D. , Evans, J. L. F. , & Curtis, G. A. (1993). The spread of Aedes albopictus in Florida. American Entomologist, 39, 163–173.

[ece39206-bib-0078] Omlin, F. X. , Carlson, J. C. , Ogbunugafo, C. B. , & Hassanali, A. (2007). Anopheles gambiae exploits the treehole ecosystem in western Kenya: A new urban malaria risk? American Journal of Tropical Medicine and Hygiene, 77, 264–269. 18165501

[ece39206-bib-0079] Paradise, C. J. (1998). Colonization and development of insects in simulated treehole habitats with distinct resource and pH regimes. Ecoscience, 5, 39–45.

[ece39206-bib-0080] Paradise, C. J. (1999). Interactive effects of resources and a processing chain interaction in Treehole habitats. Oikos, 85, 529–535.

[ece39206-bib-0081] Paradise, C. J. (2004). Relationship of water and leaf litter variability to insects inhabiting treeholes. Journal of the North American Benthological Society, 23, 793–805.

[ece39206-bib-0082] Paradise, C. J. , Blue, J. D. , Burkhart, J. Q. , Goldberg, J. , Harshaw, L. , Hawkins, K. D. , Kegan, B. , Krentz, T. , Smith, L. , & Villalpando, S. (2008). Local and regional factors influence the structure of treehole metacommunities. BMC Ecology, 8, 1–16. 19099587 10.1186/1472-6785-8-22 PMC2628885

[ece39206-bib-0083] Paradise, C. J. , & Dunson, W. A. (1997). Effects of water cations on Treehole insect communities. Annals of the Entomological Society of America, 90, 798–805.

[ece39206-bib-0084] Pawlowski, J. , Apothéloz‐Perret‐Gentil, L. , & Altermatt, F. (2020). Environmental DNA: What's behind the term? Clarifying the terminology and recommendations for its future use in biomonitoring. Molecular Ecology, 29, 4258–4264. 32966665 10.1111/mec.15643

[ece39206-bib-0085] Pawlowski, J. , Kelly‐Quinn, M. , Altermatt, F. , Apothéloz‐Perret‐Gentil, L. , Beja, P. , Boggero, A. , Borja, A. , Bouchez, A. , Cordier, T. , Domaizon, I. , Feio, M. J. , Filipe, A. F. , Fornaroli, R. , Graf, W. , Herder, J. , van der Hoorn, B. , Iwan Jones, J. , Sagova‐Mareckova, M. , Moritz, C. , … Kahlert, M. (2018). The future of biotic indices in the ecogenomic era: Integrating (e)DNA metabarcoding in biological assessment of aquatic ecosystems. Science of the Total Environment, 637‐638, 1295–1310. 10.1016/j.scitotenv.2018.05.002 29801222

[ece39206-bib-0086] Petermann, J. S. , Roberts, A. L. , Hemmerling, C. , Bajerski, F. , Pascual, J. , Overmann, J. , Weisser, W. W. , Ruess, L. , & Gossner, M. (2020). Direct and indirect effects of forest management on tree‐hole inhabiting aquatic organisms and their functional traits. Science of the Total Environment, 704, 135418. 31896218 10.1016/j.scitotenv.2019.135418

[ece39206-bib-0087] Petermann, J. S. , Rohland, A. , Sichardt, N. , Lade, P. , Guidetti, B. , Weisser, W. W. , & Gossner, M. (2016). Forest management intensity affects aquatic communities in artificial tree holes. PLoS One, 11, e0155549. 27187741 10.1371/journal.pone.0155549 PMC4871352

[ece39206-bib-0088] Pimm, S. L. , & Kitching, R. L. (1987). The determinants of food chain lengths. Oikos, 50, 302–307.

[ece39206-bib-0089] Polhemus, J. T. (1999). Two new species of Microvelia from Treeholes, with notes on other container‐inhabiting veliid species (Heteroptera: Veliidae). Journal of the New York Entomological Society, 107, 31–37.

[ece39206-bib-0090] Ponnusamy, L. , Xu, N. , Stav, G. , Wesson, D. M. , Schal, C. , & Apperson, C. S. (2008). Diversity of bacterial communities in container habitats of mosquitoes. Microbial Ecology, 56, 593–603. 18373113 10.1007/s00248-008-9379-6 PMC2904961

[ece39206-bib-0091] Ptatscheck, C. , & Traunspurger, W. (2014). The meiofauna of artificial water‐filled tree holes: Colonization and bottom‐up effects. Aquatic Ecology, 48, 285–295.

[ece39206-bib-0092] Ptatscheck, C. , & Traunspurger, W. (2015). Meio‐ and macrofaunal communities in artificial water‐filled tree holes: Effects of seasonality, physical and chemical parameters, and availability of food resources. PLoS One, 10, e0133447. 26284811 10.1371/journal.pone.0133447 PMC4540321

[ece39206-bib-0093] R Development Core Team . (2016). R: A language and environment for statistical computing. R Foundation for Statistical Computing.

[ece39206-bib-0094] Rohnert, U. (1950). Wassererfüllte Baumhöhlen und ihre Besiedlung: ein Beitrag zur Fauna dendrolimnetica. Archiv für Hydobiologie, 44, 472–518.

[ece39206-bib-0095] Sanchez, E. , & Liria, J. (2009). Relative abundance and temporal variation of macroinvertebrates in a Venezuelan cloud forest habitat. International Journal of Tropical Insect Science, 29, 3–10.

[ece39206-bib-0096] Sarremejane, R. , Cid, N. , Datry, T. , Stubbington, R. , Alp, M. , Cañedo‐Argüelles, M. , Cordero‐Rivera, A. , Csabai, Z. , Gutiérrez‐Cánovas, C. , Heino, J. , Forcellini, M. , Millán, A. , Paillex, A. , Pařil, P. , Polášek, M. , de Figueroa, J. M. T. , Usseglio‐Polatera, P. , Zamora‐Muñoz, C. , & Bonada, N. (2020). DISPERSE: A trait database to assess the dispersal potential of aquatic macroinvertebrates. Scientific Data, 7(1), 386. 33177529 10.1038/s41597-020-00732-7 PMC7658241

[ece39206-bib-0097] Schaffner, F. , Kaufmann, C. , Hegglin, D. , & Mathis, A. (2009). The invasive mosquito Aedes japonicus in Central Europe. Medical and Veterinary Entomology, 23, 448–451. 19941611 10.1111/j.1365-2915.2009.00825.x

[ece39206-bib-0098] Scherer‐Lorenzen, M. , Gessner, M. O. , Beisner, B. E. , Messier, C. , Paquette, A. , Petermann, J. S. , Soininen, J. , & Nock, C. A. (2022). Pathways for cross‐boundary effects of biodiversity on ecosystem functioning. Trends in Ecology and Evolution, 37, 454–467. 35065823 10.1016/j.tree.2021.12.009

[ece39206-bib-0099] Schmidl, J. , Sulzer, P. , & Kitching, R. L. (2008). The insect assemblage in water filled tree‐holes in a European temperate deciduous forest: Community composition reflects structural, trophic and physicochemical factors. Hydrobiologia, 598, 285–303.

[ece39206-bib-0100] Schulz, G. , Siqueira, T. , Stefan, G. , & de Oliveira Roque, F. (2012). Passive and active dispersers respond similarly to environmental and spatial processes: An example from metacommunity dynamics of tree hole invertebrates. Fundamental and Applied Limnology/Archiv für Hydrobiologie, 181, 315–326.

[ece39206-bib-0101] Sciullo, L. , & Kolasa, J. (2012). Linking local community structure to the dispersal of aquatic invertebrate species in a rock pool metacommunity. Community Ecology, 13, 203–212.

[ece39206-bib-0102] Smith, L. M. , Blue, J. D. , Carlson, J. , Metz, G. , Haywood, J. , Bush, D. , & Paradise, C. J. (2009). Density‐dependent predation of a dominant species does not facilitate increased diversity in Treeholes. The Open Ecology Journal, 2, 91–99.

[ece39206-bib-0103] Soininen, J. , Bartels, P. , Heino, J. , Luoto, M. , & Hillebrand, H. (2015). Toward more integrated ecosystem research in aquatic and terrestrial environments. Bioscience, 65, 174–182.

[ece39206-bib-0104] Sridhar, K. R. , Karamchand, K. S. , & Seena, S. (2013). Fungal assemblage and leaf litter decomposition in riparian tree holes and in a coastal stream of the south‐West India. Mycology, 4, 118–124.

[ece39206-bib-0105] Srivastava, D. S. (2005). Do local processes scale to global patterns? The role of drought and the species pool in determining treehole insect diversity. Oecologia, 145, 205–215. 15891844 10.1007/s00442-005-0061-0

[ece39206-bib-0106] Srivastava, D. S. , Céréghino, R. , Trzcinski, M. K. , MacDonald, A. A. M. , Marino, N. A. C. , Mercado, D. A. , Leroy, C. , Corbara, B. , Romero, G. Q. , Farjalla, V. F. , Barberis, I. M. , Dézerald, O. , Hammill, E. , Atwood, T. B. , Piccoli, G. C. O. , Ospina‐Bautista, F. , Carrias, J.‐F. , Leal, J. S. , Montero, G. , … Campos, A. B. A. (2020). Ecological response to altered rainfall differs across the neotropics. Ecology, 101, e02984. 31958151 10.1002/ecy.2984

[ece39206-bib-0107] Srivastava, D. S. , Kolasa, J. , Bengtsson, J. , Gonzalez, A. , Lawler, S. P. , Miller, T. E. , Munguia, P. , Romanuk, T. , Schneider, D. C. , & Trzcinski, M. K. (2004). Are natural microcosms useful model systems for ecology? Trends in Ecology & Evolution, 19, 379–384. 16701289 10.1016/j.tree.2004.04.010

[ece39206-bib-0108] Srivastava, D. S. , & Lawton, J. H. (1998). Why more productive sites have more species: An experimental test of theory using tree‐hole communities. American Naturalist, 152, 510–529. 10.1086/286187 18811361

[ece39206-bib-0109] Srivastava, D. S. , MacDonald, A. A. M. , Pillar, V. D. , Kratina, P. , Debastiani, V. J. , Guzman, L. M. , Trzcinski, M. K. , Dézerald, O. , Barberis, I. M. , de Omena, P. M. , Romero, G. Q. , Ospina‐Bautista, F. , Marino, N. A. C. , Leroy, C. , Farjalla, V. F. , Richardson, B. A. , Gonçalves, A. Z. , Corbara, B. , Petermann, J. S. , … Céréghino, R. (2022). Geographical variation in the trait‐based assembly patterns of multitrophic invertebrate communities. Functional Ecology. 10.1111/1365-2435.14096

[ece39206-bib-0110] Strauß, L. , R. Faustino de Lima , F. Riesbeck , and M.‐O. Rödel . 2018. São Tomé Island endemic treefrogs (*Hyperolius* spp.) and land‐use intensification: A tale of Hope and caution. Tropical conservation Science 11, 1–14. 10.1177/1940082918776434

[ece39206-bib-0111] Suter, T. , Crespo, M. M. , de Oliveira, M. F. , de Oliveira, T. S. A. , de Melo‐Santos, M. A. V. , de Oliveira, C. M. F. , Ayres, C. F. J. , Barbosa, R. M. R. , Araújo, A. P. , Regis, L. N. , Flacio, E. , Engeler, L. , Müller, P. , & Silva‐Filha, M. H. N. L. (2017). Insecticide susceptibility of *Aedes albopictus* and *ae. Aegypti* from Brazil and the swiss‐Italian border region. Parasites & Vectors, 10(431), 431. 28927441 10.1186/s13071-017-2364-5 PMC5606125

[ece39206-bib-0112] Tate, P. (1935). The larva of *Phaonia mirabilis* Ringdahl, predatory on mosquito larvae (Diptera, Anthomyidae). Parasitology, 27, 556–560.

[ece39206-bib-0113] Thienemann, A. (1934). Die Tierwelt der tropischen Pflanzengewasser. Archiv für Hydrobiologie Supplement, 13, 1–91.

[ece39206-bib-0114] Thomas, S. M. , Tjaden, N. B. , Frank, C. , Jaeschke, A. , Zipfel, L. , Wagner‐Wiening, C. , Faber, M. , Beierkuhnlein, C. , & Stark, K. (2018). Areas with high Hazard potential for autochthonous transmission of Aedes albopictus‐associated arboviruses in Germany. International Journal of Environmental Research and Public Health, 15, 1270. 10.3390/ijerph15061270 PMC6025521 29914102

[ece39206-bib-0115] Udvardy, M. 1975. A Classification of the Biogeographical Provinces of the World. IUCN Occasional Paper No. 18. International Union for Conservation of Nature and Natural Resources.

[ece39206-bib-0116] Verdonschot, R. C. M. , Febria, C. M. , & Williams, D. D. (2008). Fluxes of dissolved organic carbon, other nutrients and microbial communities in a water‐filled treehole ecosystem. Hydrobiologia, 596, 17–30.

[ece39206-bib-0117] Viechtbauer, W. (2010). Conducting meta‐analyses in R with the metafor package. Journal of Statistical Software, 36, 1–48.

[ece39206-bib-0118] von Brandt, A. (1934). Untersuchungen in Baumhöhlengewässern auf *Fagus silvatica* . Archiv für Hydobiologie, 27, 546–563.

[ece39206-bib-0119] Walker, E. , O'Meara, G. , & Morgan, W. T. (1996). Bacterial abundance in larval habitats of Aedes albopictus (diptera: Culicidae) in a Florida cemetery. Journal of Vector Ecology, 21, 173–177.

[ece39206-bib-0120] Walker, E. D. , Kaufman, M. G. , & Merritt, R. W. (2010). An acute trophic cascade among microorganisms in the tree hole ecosystem following removal of omnivorous mosquito larvae. Community Ecology, 11, 171–178. 25342946 10.1556/ComEc.11.2010.2.5 PMC4204208

[ece39206-bib-0121] Walker, E. D. , & Merritt, R. W. (1988). The significance of leaf detritus to mosquito (Diptera: Culicidae) productivity from Treeholes. Environmental Entomology, 17, 199–206.

[ece39206-bib-0122] Washburn, J. O. , Anderson, J. R. , & Mercer, D. R. (1989). Emergence characteristics of Aedes sierrensis (Diptera: Culicidae) from California Treeholes with particular reference to parasite loads. Journal of Medical Entomology, 26, 173–182. 2724315 10.1093/jmedent/26.3.173

[ece39206-bib-0123] Westby, K. M. , Juliano, S. A. , & Medley, K. A. (2020). Aedes albopictus (Diptera: Culicidae) has not become the dominant species in artificial container habitats in a temperate Forest more than a decade after establishment. Journal of Medical Entomology, 58, 950–955. 10.1093/jme/tjaa215 PMC8244635 33073848

[ece39206-bib-0124] Weterings, R. , Umponstira, C. , & Buckley, H. L. (2014). Container‐breeding mosquitoes and predator community dynamics along an urban‐forest gradient: The effects of habitat type and isolation. Basic and Applied Ecology, 15, 486–495.

[ece39206-bib-0125] Wong, M. K. L. , Guénard, B. , & Lewis, O. T. (2019). Trait‐based ecology of terrestrial arthropods. Biological Reviews, 94, 999–1022. 30548743 10.1111/brv.12488 PMC6849530

[ece39206-bib-0126] Woodcock, S. , Van Der Gast, C. J. , Bell, T. , Lunn, M. , Curtis, T. P. , Head, I. M. , & Sloan, W. T. (2007). Neutral assembly of bacterial communities. FEMS Microbiology Ecology, 62, 171–180. 17937674 10.1111/j.1574-6941.2007.00379.x

[ece39206-bib-0127] Yanoviak, S. (1999a). Distribution and abundance of *Microvelia cavicola* Polhemus (Heteroptera: Veliidae) on Barro Colorado Island, Panama. Journal of the New York Entomological Society, 107, 38–45.

[ece39206-bib-0128] Yanoviak, S. P. (1999b). Community structure in water‐filled tree holes of Panama: Effects of hole height and size. Selbyana, 20, 106–115.

[ece39206-bib-0129] Yanoviak, S. P. (2001a). The macrofauna of water‐filled tree holes on Barro Colorado Island, Panama. Biotropica, 33, 110–120.

[ece39206-bib-0130] Yanoviak, S. P. (2001b). Predation, resource availability, and community structure in neotropical water‐filled tree holes. Oecologia, 126, 125–133. 28547431 10.1007/s004420000493

[ece39206-bib-0131] Yanoviak, S. P. , Lounibos, L. P. , & Weaver, S. C. (2006). Land use affects macroinvertebrate community composition in phytotelmata in the Peruvian Amazon. Annals of the Entomological Society of America, 99, 1172–1181.

[ece39206-bib-0132] Yee, D. A. , & Juliano, S. A. (2007). Abundance matters: A field experiment testing the more individuals hypothesis for richness‐productivity relationships. Oecologia, 153, 153–162. 17401581 10.1007/s00442-007-0707-1 PMC2040027

[ece39206-bib-0133] Yee, D. A. , Yee, S. H. , Kneitel, J. M. , & Juliano, S. A. (2007). Richness‐productivity relationships between trophic levels in a detritus‐based system: Significance of abundance and trophic linkage. Oecologia, 154, 377–385. 17713787 10.1007/s00442-007-0837-5 PMC2638089

