# Supplementary figures and images for: Aquatic islands in the sky: 100 years of research on water‐filled tree holes
Source: Ecol Evol. 2022 Aug 12;12(8):e9206. doi: 10.1002/ece3.9206 (PMC9374645; doi:10.1002/ece3.9206)

**A**

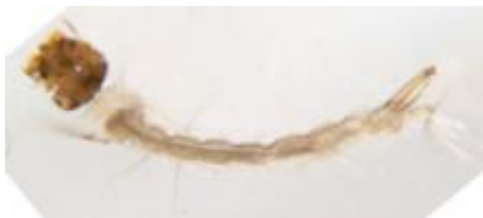

**B**

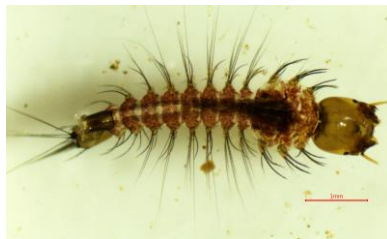

**C**

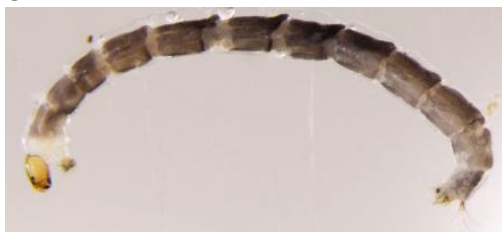

**D**

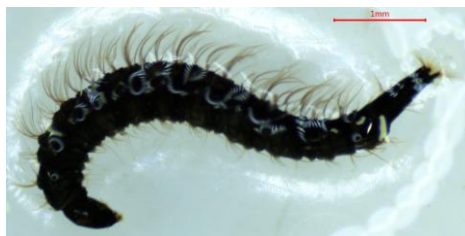

**E**

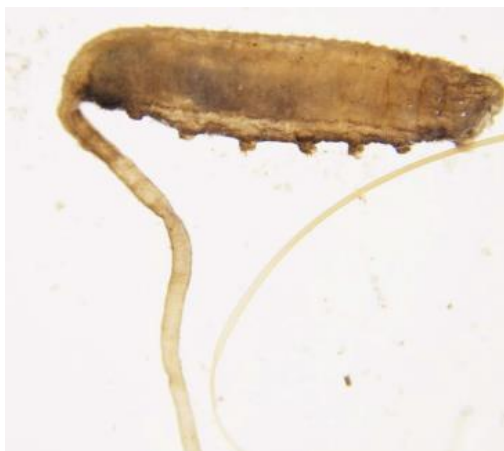

**F**

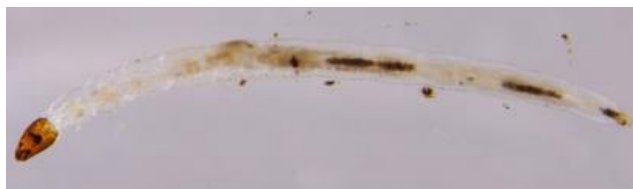

**G**

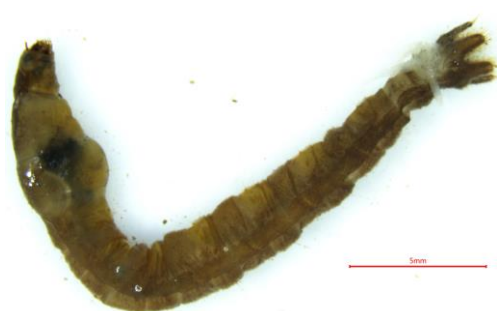

**H**

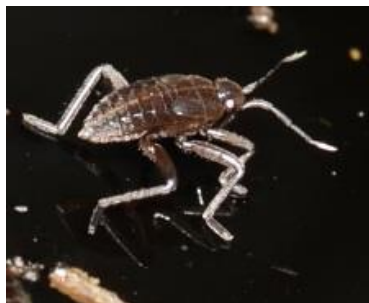

**I**

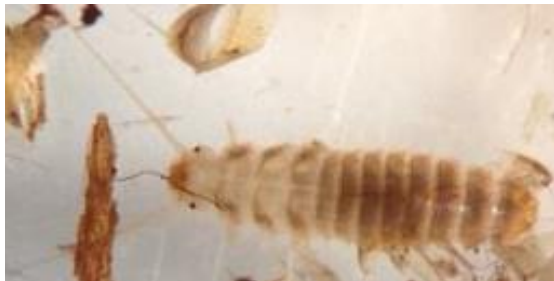

**J**

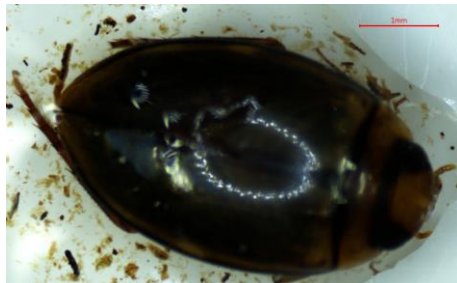

K

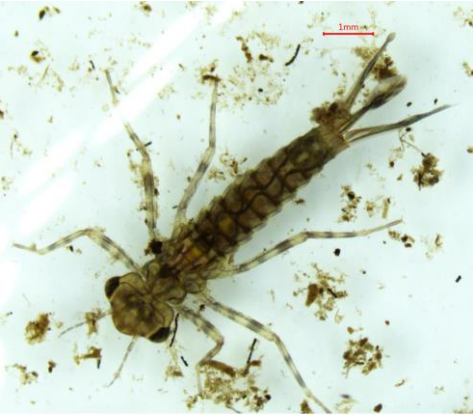

L

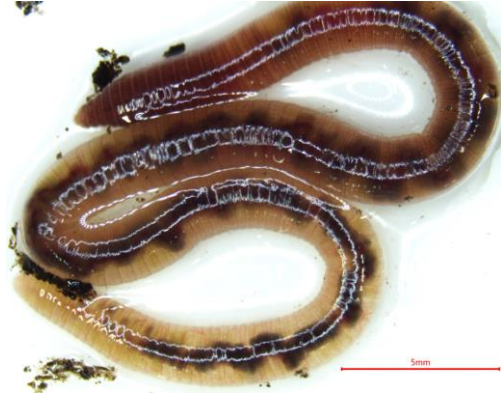

M

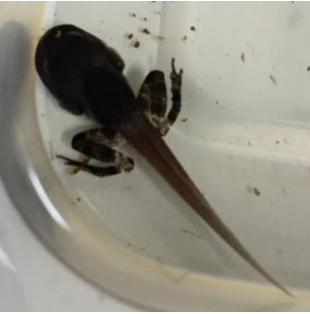

N

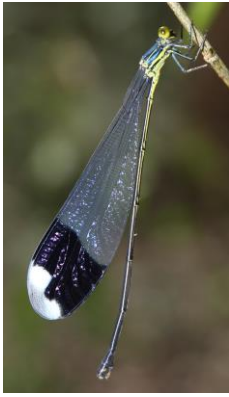

O

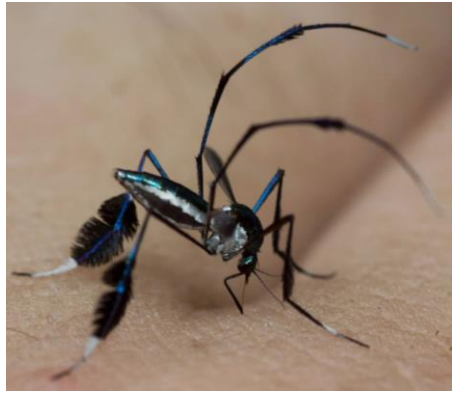

Supplement: Supplementary file 1 — Figure S1 [file ECE3-12-e9206-s002.pdf]

A

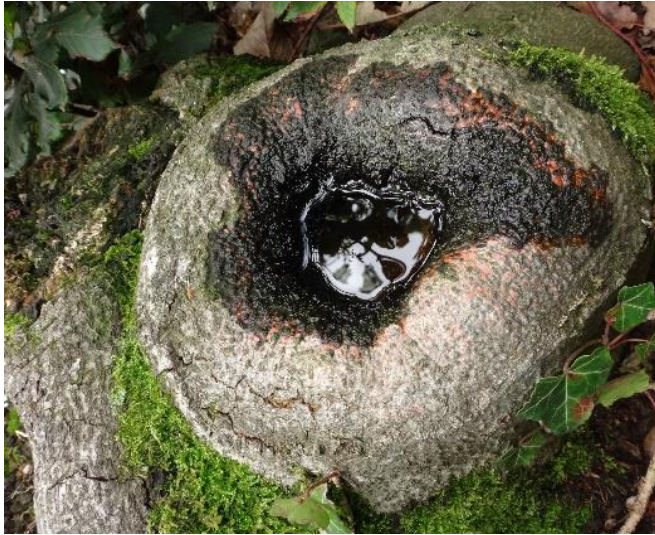

B

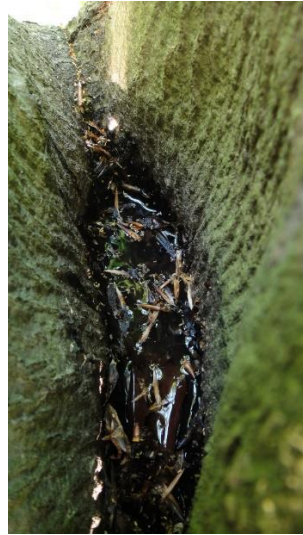

C

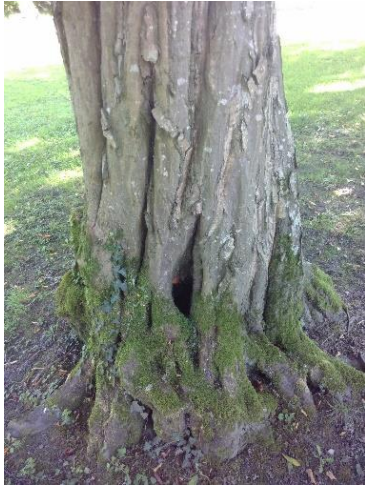

D

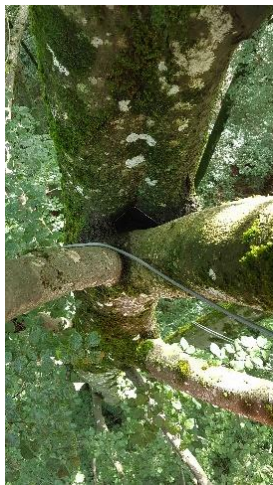

E

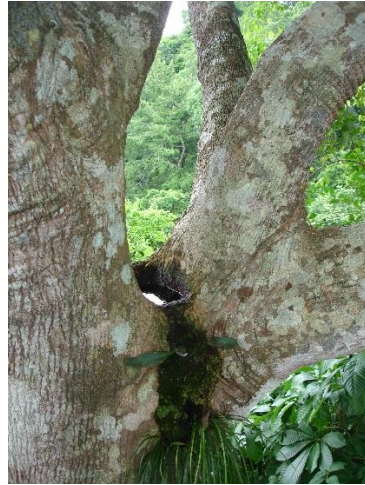

F

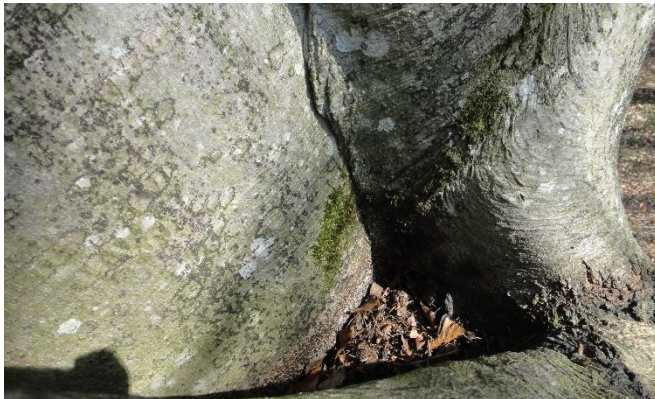

G

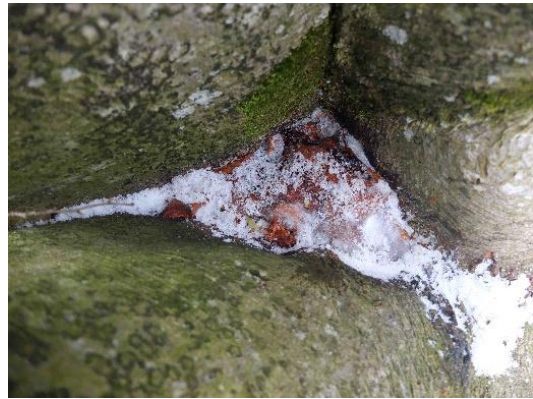

H

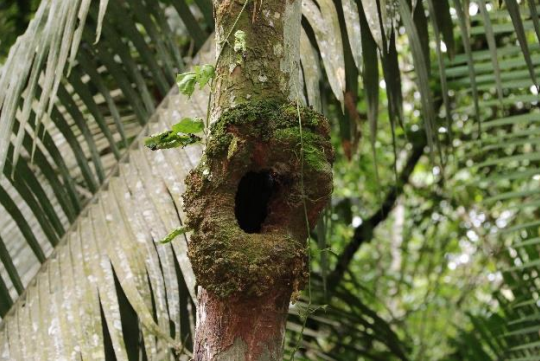

I

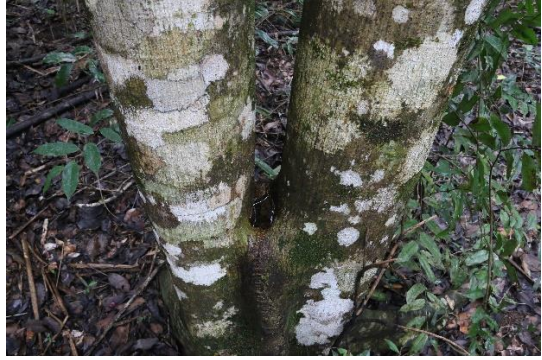

J

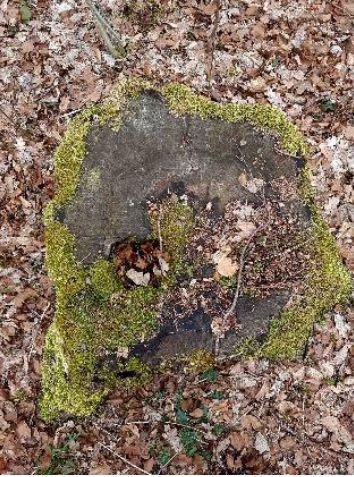

K

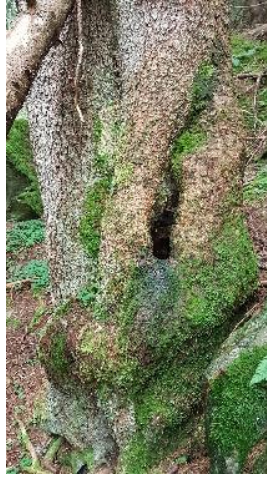

L

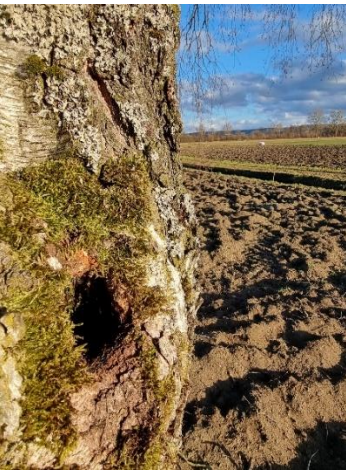

M

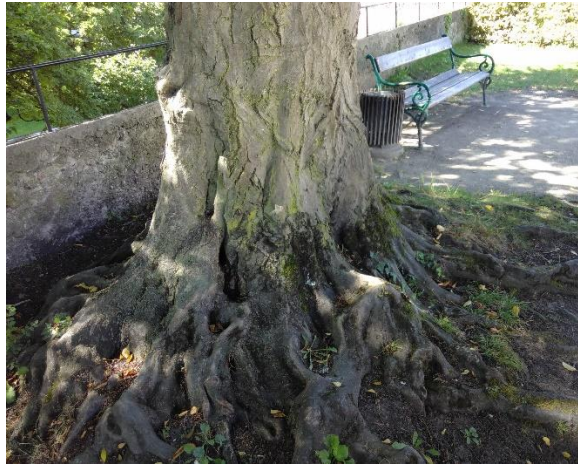

Supplement: Supplementary file 2 — Figure S2 [file ECE3-12-e9206-s001.pdf]

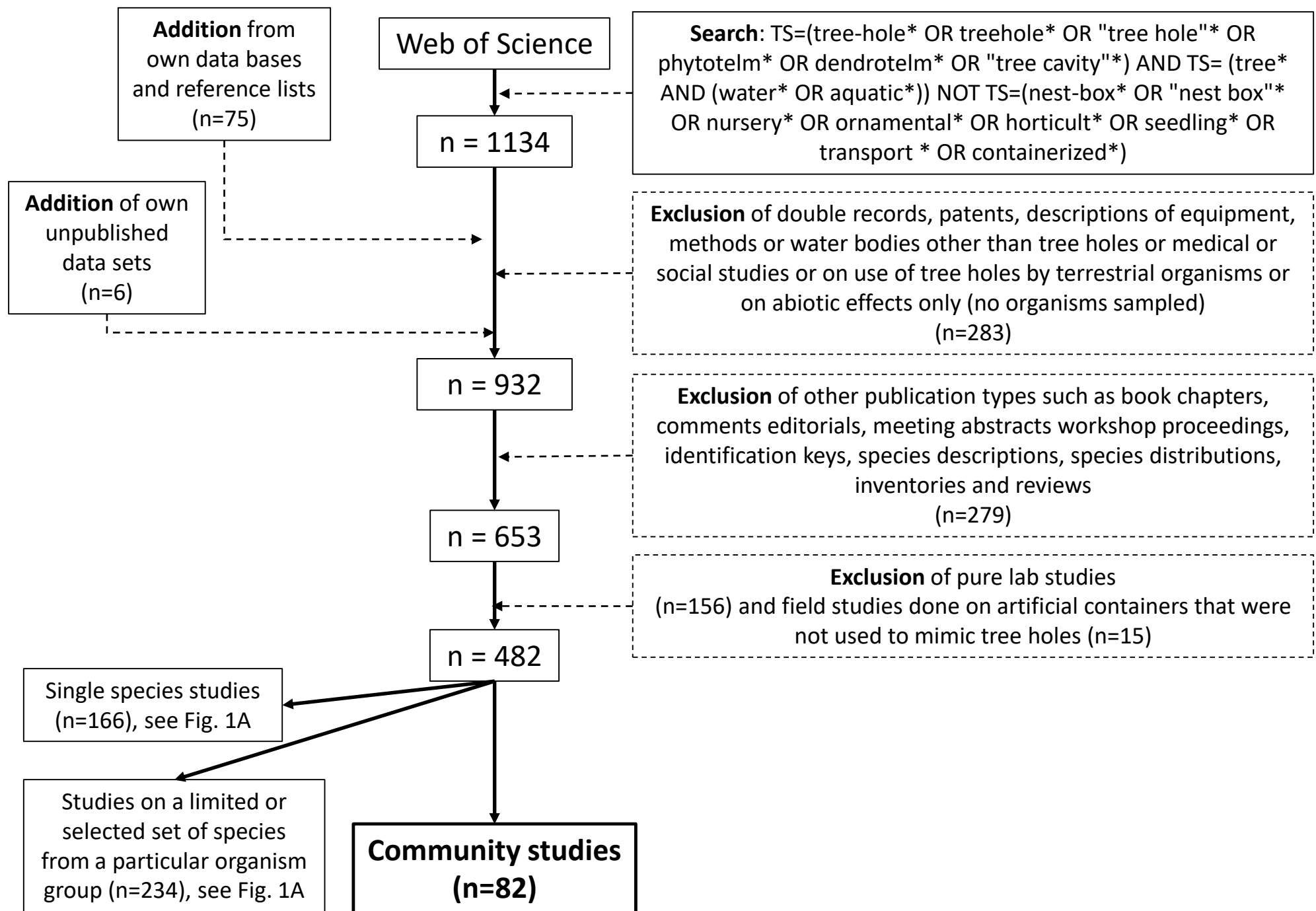

Supplement: Supplementary file 3 — Figure S3 [file ECE3-12-e9206-s006.pdf]

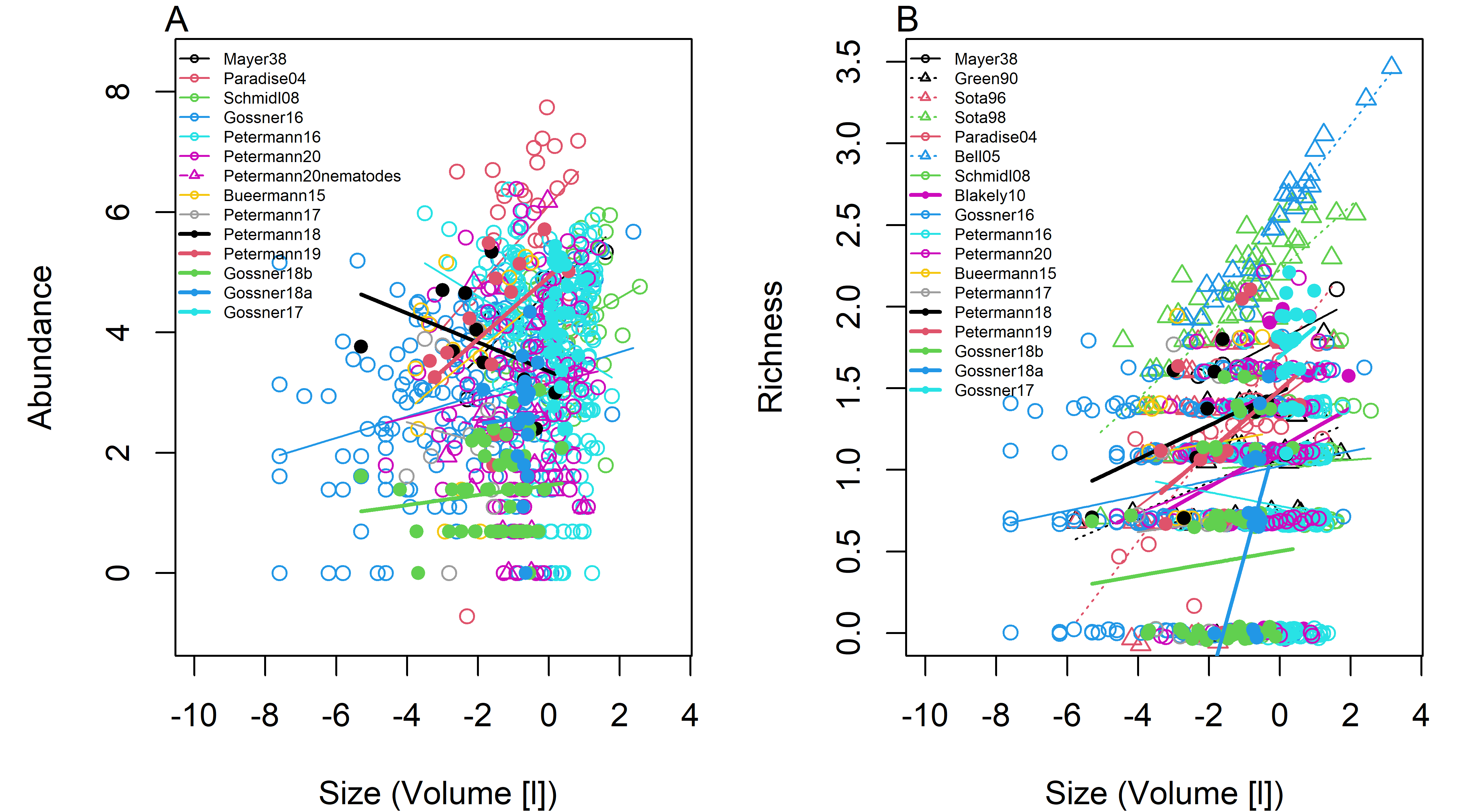

Supplement: Supplementary file 4 — Figure S4 [file ECE3-12-e9206-s004.tiff]

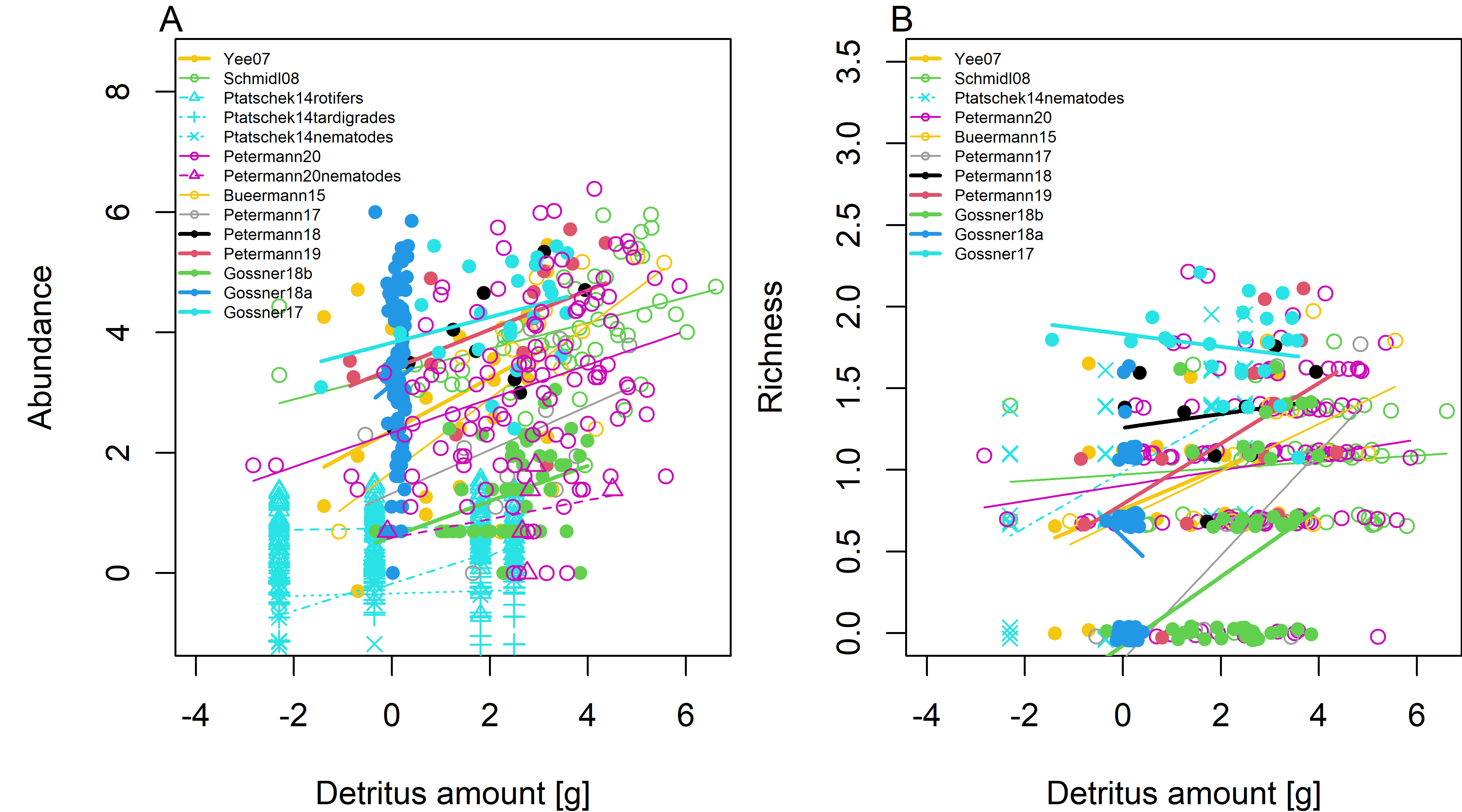

Supplement: Supplementary file 5 — Figure S5 [file ECE3-12-e9206-s005.tiff]

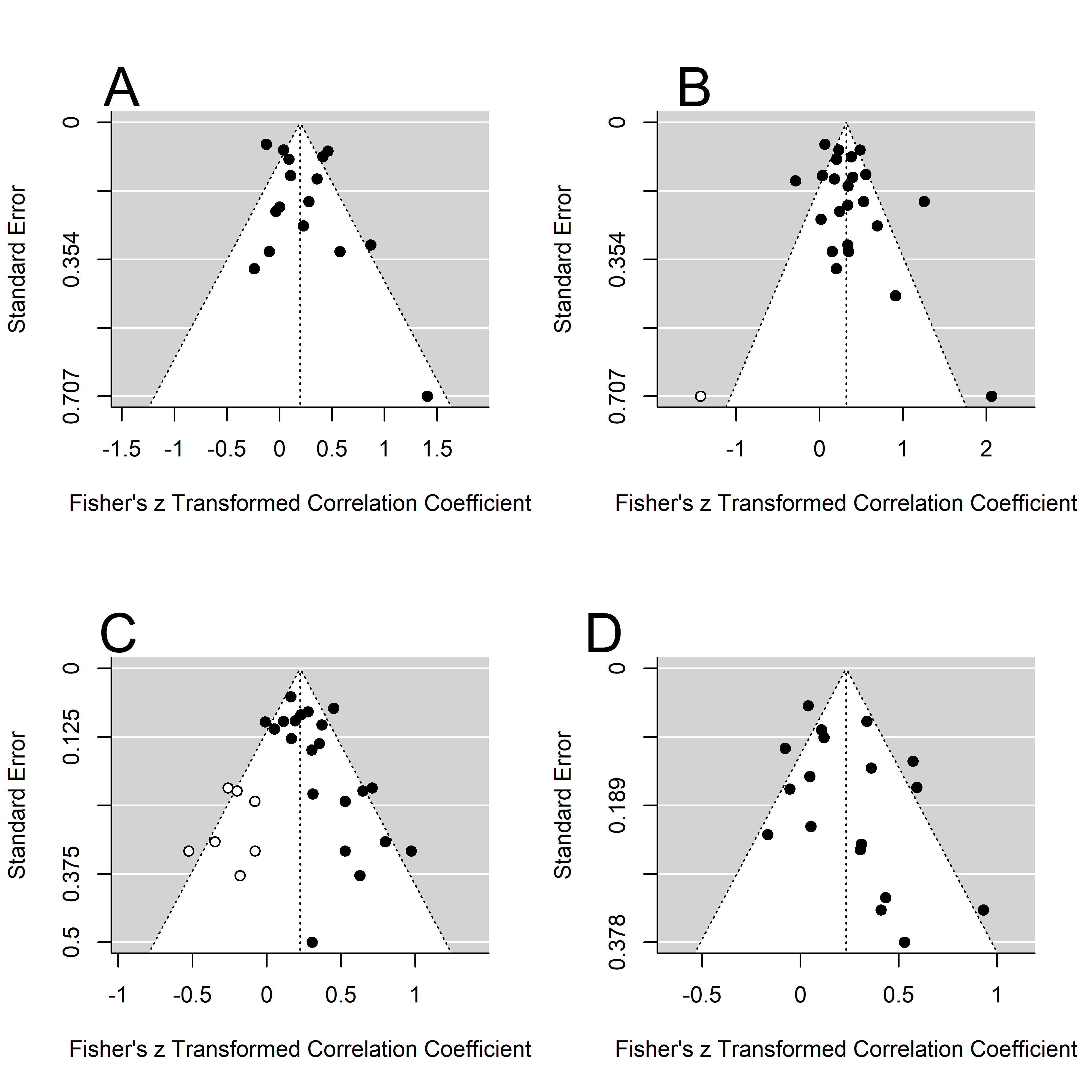

Supplement: Supplementary file 6 — Figure S6 [file ECE3-12-e9206-s007.tiff]
