# Supplementary material for: Aquatic islands in the sky: 100 years of research on water‐filled tree holes
Source: Ecol Evol. 2022 Aug 12;12(8):e9206. doi: 10.1002/ece3.9206 (PMC9374645; doi:10.1002/ece3.9206)
Supplement: Supplementary file 7 — Table S1‐S2 [file ECE3-12-e9206-s003.docx]

**Table S1:** List of community studies (n=82) and information such as latitude, longitude and climate of the study site, whether the study included natural and/or artificial tree holes, whether it included an experimental and/or an observational set-up and which organisms were sampled. For multi-site studies, longitude and latitude of one study site is shown only (as used in the map in Fig. 2). Most of these multi-site studies sampled in study sites that were close together on a global scale (<300km distance). The grouping of organisms is as defined and used in Fig. 1c. References of all studies can be found in the reference list below.

| **Year** | **First author** | **Latitude** | **Longitude** | **Climate** | **Natural** | **Artificial** | **Experiment** | **Observation** | **Insects** | **Mites** | **Polychaetes** | **Gastropods** | **Micro-crustaceans** | **Crabs** | **Amphibians** | **Protists** | **Bacteria** | **Fungi** | **Nematodes** | **Rotifers** | **Tardigrades** |
| --- | --- | --- | --- | --- | --- | --- | --- | --- | --- | --- | --- | --- | --- | --- | --- | --- | --- | --- | --- | --- | --- |
| 1927 | Keilin | 52.201746 | 0.122326 | temperate | 1 | 0 | 0 | 1 | 1 | 0 | 0 | 0 | 0 | 0 | 0 | 0 | 0 | 0 | 0 | 0 | 0 |
| 1934 | Thienemann | -7.796049 | 111.632706 | tropical | 1 | 0 | 0 | 1 | 1 | 1 | 1 | 0 | 1 | 0 | 1 | 1 | 0 | 0 | 1 | 1 | 0 |
| 1934 | von Brandt | 54.2500 | 19.5833 | temperate | 1 | 0 | 0 | 1 | 1 | 0 | 1 | 0 | 1 | 0 | 1 | 1 | 0 | 0 | 1 | 1 | 0 |
| 1938 | Mayer | 48.1663 | 17.0253 | temperate | 1 | 0 | 0 | 1 | 1 | 0 | 1 | 0 | 1 | 0 | 0 | 1 | 0 | 0 | 0 | 0 | 0 |
| 1940 | Lackey | 33.1568 | -87.3035 | temperate | 1 | 0 | 0 | 1 | 0 | 0 | 0 | 0 | 0 | 0 | 0 | 1 | 0 | 0 | 0 | 0 | 0 |
| 1940 | Pavisic | 45.3182 | 17.6645 | temperate | 1 | 0 | 0 | 1 | 1 | 0 | 0 | 0 | 1 | 0 | 0 | 0 | 0 | 0 | 0 | 1 | 0 |
| 1950 | Rohnert | 54.1610 | 10.7597 | temperate | 1 | 0 | 0 | 1 | 1 | 0 | 1 | 1 | 0 | 0 | 0 | 1 | 0 | 0 | 0 | 1 | 0 |
| 1958 | Snow | 34.705347 | -87.40655 | temperate | 1 | 0 | 0 | 1 | 1 | 0 | 0 | 0 | 0 | 0 | 0 | 0 | 0 | 0 | 0 | 0 | 0 |
| 1969 | Kitching | 51.7772 | -1.3089 | temperate | 1 | 0 | 0 | 1 | 1 | 0 | 0 | 0 | 1 | 0 | 0 | 0 | 0 | 0 | 0 | 0 | 0 |
| 1981 | Heaps | 39.6689 | -79.8116 | temperate | 0 | 1 | 0 | 1 | 1 | 0 | 0 | 0 | 0 | 0 | 0 | 0 | 0 | 0 | 0 | 0 | 0 |
| 1982 | Kitching | -28.2167 | 153.1500 | subtropical | 1 | 0 | 0 | 1 | 1 | 1 | 0 | 0 | 0 | 0 | 1 | 0 | 0 | 0 | 0 | 0 | 0 |
| 1987 | Kitching a | 0.5588 | 123.7051 | tropical | 1 | 0 | 0 | 1 | 1 | 0 | 0 | 1 | 0 | 0 | 0 | 0 | 0 | 0 | 0 | 0 | 0 |
| 1987 | Kitching b | -28.2167 | 153.1500 | subtropical | 1 | 0 | 0 | 1 | 1 | 1 | 0 | 0 | 0 | 0 | 1 | 0 | 0 | 0 | 0 | 0 | 0 |
| 1987 | Pimm | -28.2000 | 153.0000 | subtropical | 0 | 1 | 1 | 0 | 1 | 0 | 0 | 0 | 0 | 0 | 1 | 0 | 0 | 0 | 0 | 0 | 0 |
| 1988 | Woodward | 38.9248 | -122.9454 | temperate | 1 | 0 | 0 | 1 | 1 | 0 | 0 | 0 | 0 | 0 | 0 | 0 | 0 | 0 | 0 | 0 | 0 |
| 1989 | Copeland | 41.5361 | -86.3598 | temperate | 1 | 0 | 0 | 1 | 1 | 0 | 0 | 0 | 0 | 0 | 0 | 0 | 0 | 0 | 0 | 0 | 0 |
| 1990 | Green | 54.5199 | -1.5791 | temperate | 1 | 0 | 0 | 1 | 1 | 1 | 1 | 0 | 0 | 0 | 0 | 1 | 0 | 0 | 1 | 1 | 0 |
| 1990 | Jenkins | -28.2167 | 153.1500 | subtropical | 1 | 0 | 1 | 0 | 1 | 1 | 0 | 0 | 0 | 0 | 1 | 0 | 0 | 0 | 0 | 0 | 0 |
| 1990 | Kitching | -5.0634 | 145.7853 | tropical | 1 | 0 | 0 | 1 | 1 | 1 | 1 | 0 | 1 | 0 | 0 | 0 | 0 | 0 | 0 | 0 | 0 |
| 1992 | Jenkins | -30 | 152 | subtropical | 0 | 1 | 1 | 0 | 1 | 1 | 0 | 0 | 0 | 0 | 0 | 0 | 0 | 0 | 0 | 0 | 0 |
| 1996 | Barrera | 40.6949 | -77.8198 | temperate | 1 | 0 | 0 | 1 | 1 | 1 | 0 | 0 | 0 | 0 | 0 | 0 | 0 | 0 | 0 | 0 | 0 |
| 1996 | Kitching | 4.5333 | 115.1500 | tropical | 1 | 0 | 0 | 1 | 1 | 0 | 1 | 0 | 1 | 0 | 1 | 0 | 0 | 0 | 0 | 0 | 0 |
| 1996 | Sota | 34.4938 | 129.3676 | temperate | 1 | 0 | 0 | 1 | 1 | 0 | 1 | 0 | 0 | 0 | 0 | 0 | 0 | 0 | 1 | 0 | 0 |
| 1997 | Paradise | 40.8766 | -77.8367 | temperate | 0 | 1 | 1 | 1 | 1 | 0 | 0 | 0 | 0 | 0 | 0 | 0 | 0 | 0 | 0 | 0 | 0 |
| 1998 | Paradise | 40.7583 | -78.2833 | temperate | 0 | 1 | 1 | 0 | 1 | 0 | 0 | 0 | 0 | 0 | 0 | 0 | 0 | 0 | 0 | 0 | 0 |
| 1998 | Sota | 24.3254 | 123.8141 | subtropical, temperate | 1 | 0 | 0 | 1 | 1 | 0 | 1 | 0 | 1 | 0 | 1 | 0 | 0 | 0 | 1 | 0 | 0 |
| 1998 | Srivastava | 51.4120 | -0.6407 | temperate | 0 | 1 | 1 | 0 | 1 | 0 | 0 | 0 | 0 | 0 | 0 | 0 | 0 | 0 | 0 | 0 | 0 |
| 1999 | Copeland | 1.1800 | 37.3500 | tropical | 1 | 0 | 0 | 1 | 1 | 0 | 0 | 0 | 0 | 0 | 0 | 0 | 0 | 0 | 0 | 0 | 0 |
| 1999 | Yanoviak a | 9.1521 | -79.8465 | tropical | 1 | 1 | 1 | 1 | 1 | 0 | 0 | 0 | 0 | 0 | 1 | 0 | 0 | 0 | 0 | 0 | 0 |
| 1999 | Yanoviak b | 9.1521 | -79.8465 | tropical | 1 | 1 | 1 | 1 | 1 | 0 | 0 | 0 | 0 | 0 | 0 | 0 | 0 | 0 | 0 | 0 | 0 |
| 2001 | Yanoviak a | 9.1521 | -79.8465 | tropical | 0 | 1 | 1 | 0 | 1 | 0 | 1 | 0 | 0 | 0 | 0 | 0 | 0 | 0 | 0 | 0 | 0 |
| 2001 | Yanoviak c | 9.1521 | -79.8465 | tropical | 1 | 1 | 1 | 0 | 1 | 0 | 0 | 0 | 0 | 0 | 1 | 0 | 0 | 0 | 0 | 0 | 0 |
| 2001 | Yanoviak b | 9.1521 | -79.8465 | tropical | 1 | 1 | 0 | 1 | 1 | 1 | 1 | 0 | 1 | 0 | 1 | 0 | 0 | 0 | 1 | 0 | 0 |
| 2003 | Gonczol | 46.5236 | 16.7291 | temperate | 1 | 0 | 0 | 1 | 0 | 0 | 0 | 0 | 0 | 0 | 0 | 0 | 0 | 1 | 0 | 0 | 0 |
| 2003 | Taylor | -43.8115 | 173.0316 | temperate | 1 | 0 | 0 | 1 | 1 | 0 | 0 | 0 | 0 | 0 | 0 | 0 | 0 | 0 | 0 | 0 | 0 |
| 2004 | Devetter | 48.9720 | 13.6331 | temperate | 1 | 0 | 0 | 1 | 1 | 1 | 0 | 0 | 1 | 0 | 0 | 0 | 0 | 0 | 1 | 1 | 1 |
| 2004 | Paradise | 40.7500 | -78.2833 | temperate | 1 | 0 | 0 | 1 | 1 | 0 | 0 | 0 | 0 | 0 | 0 | 0 | 0 | 0 | 0 | 0 | 0 |
| 2005 | Bell | 51.7772 | -1.3089 | temperate | 1 | 0 | 0 | 1 | 0 | 0 | 0 | 0 | 0 | 0 | 0 | 0 | 1 | 0 | 0 | 0 | 0 |
| 2005 | Cumberlidge | -15.6957 | 49.9763 | tropical | 1 | 0 | 0 | 1 | 0 | 0 | 0 | 0 | 0 | 1 | 0 | 0 | 0 | 0 | 0 | 0 | 0 |
| 2005 | Srivastava | 51.4167 | -0.5833 | temperate | 1 | 0 | 0 | 1 | 1 | 0 | 0 | 0 | 0 | 0 | 0 | 0 | 0 | 0 | 0 | 0 | 0 |
| 2006 | Harlan | 35.5103 | -80.8300 | temperate | 0 | 1 | 1 | 0 | 1 | 0 | 0 | 0 | 0 | 0 | 0 | 0 | 0 | 0 | 0 | 0 | 0 |
| 2006 | Yanoviak | -3.7500 | -73.2500 | tropical | 1 | 1 | 0 | 1 | 1 | 0 | 0 | 0 | 0 | 0 | 0 | 0 | 0 | 0 | 0 | 0 | 0 |
| 2007 | Yee | 37.1379 | -89.3430 | temperate | 1 | 0 | 0 | 1 | 1 | 0 | 0 | 0 | 0 | 0 | 0 | 1 | 0 | 0 | 0 | 0 | 0 |
| 2007 | Yee&Juliano | 40.6528 | -88.8725 | temperate | 0 | 1 | 1 | 0 | 1 | 0 | 0 | 0 | 0 | 0 | 0 | 0 | 0 | 0 | 0 | 0 | 0 |
| 2008 | Karamchand | 12.8141 | 74.9273 | tropical | 1 | 0 | 0 | 1 | 0 | 0 | 0 | 0 | 0 | 0 | 0 | 0 | 0 | 1 | 0 | 0 | 0 |
| 2008 | Kaufman | 42.7037 | -84.4650 | temperate | 1 | 0 | 1 | 0 | 0 | 0 | 0 | 0 | 0 | 0 | 0 | 0 | 1 | 1 | 0 | 0 | 0 |
| 2008 | Paradise | 35.5103 | -80.8300 | temperate | 1 | 0 | 0 | 1 | 1 | 0 | 0 | 0 | 0 | 0 | 0 | 0 | 0 | 0 | 0 | 0 | 0 |
| 2008 | Ponnusamy | 29.8437 | -90.0792 | subtropical | 1 | 0 | 0 | 1 | 0 | 0 | 0 | 0 | 0 | 0 | 0 | 0 | 1 | 0 | 0 | 0 | 0 |
| 2008 | Schmidl | 49.6675 | 11.1667 | temperate | 1 | 0 | 0 | 1 | 1 | 0 | 0 | 0 | 0 | 0 | 0 | 0 | 0 | 0 | 0 | 0 | 0 |
| 2008 | Verdonschot | 43.7817 | -79.1843 | temperate | 1 | 0 | 0 | 1 | 0 | 0 | 0 | 0 | 0 | 0 | 0 | 0 | 1 | 0 | 0 | 0 | 0 |
| 2009 | Sanchez | 10.3717 | -67.7227 | subtropical | 0 | 1 | 0 | 1 | 1 | 0 | 0 | 0 | 0 | 0 | 0 | 0 | 0 | 0 | 0 | 0 | 0 |
| 2009 | Smith | 35.5103 | -80.8300 | temperate | 1 | 1 | 1 | 1 | 1 | 0 | 0 | 0 | 0 | 0 | 0 | 0 | 0 | 0 | 0 | 0 | 0 |
| 2010 | Ager | NA | NA | temperate | 0 | 1 | 1 | 0 | 0 | 0 | 0 | 0 | 0 | 0 | 0 | 0 | 1 | 0 | 0 | 0 | 0 |
| 2010 | Bell | 51.7772 | -1.3089 | temperate | 1 | 1 | 1 | 1 | 0 | 0 | 0 | 0 | 0 | 0 | 0 | 0 | 1 | 0 | 0 | 0 | 0 |
| 2010 | Blakely | -41.7089 | 171.9609 | temperate | 0 | 1 | 1 | 0 | 1 | 0 | 0 | 0 | 0 | 0 | 0 | 0 | 0 | 0 | 0 | 0 | 0 |
| 2010 | Walker | 42.7037 | -84.4650 | temperate | 1 | 0 | 1 | 0 | 0 | 0 | 0 | 0 | 0 | 0 | 0 | 1 | 1 | 0 | 0 | 0 | 0 |
| 2012 | Blakely | -41.7089 | 171.9609 | temperate | 1 | 1 | 0 | 1 | 1 | 0 | 0 | 0 | 0 | 0 | 0 | 0 | 0 | 0 | 0 | 0 | 0 |
| 2012 | Nishadh | 11.0641 | 76.5378 | tropical | 1 | 0 | 0 | 1 | 1 | 0 | 0 | 0 | 0 | 0 | 0 | 0 | 0 | 0 | 0 | 0 | 0 |
| 2012 | Schulz | -21.1165 | -56.6527 | temperate | 1 | 0 | 0 | 1 | 1 | 0 | 1 | 0 | 1 | 0 | 0 | 0 | 0 | 0 | 0 | 0 | 0 |
| 2012 | Yee | 40.6528 | -88.8725 | temperate | 0 | 1 | 1 | 0 | 1 | 0 | 0 | 0 | 0 | 0 | 0 | 0 | 0 | 0 | 0 | 0 | 0 |
| 2014 | Ptatscheck | 52.0321 | 8.4907 | temperate | 0 | 1 | 1 | 0 | 0 | 0 | 0 | 0 | 0 | 0 | 0 | 0 | 0 | 0 | 1 | 1 | 1 |
| 2015 | Büermann | 52.51667 | 13.4 | temperate | 1 | 1 | 0 | 1 | 1 | 0 | 0 | 0 | 0 | 0 | 0 | 0 | 0 | 0 | 0 | 0 | 0 |
| 2015 | Khazan | 18.5326 | -95.1629 | tropical | 0 | 1 | 0 | 1 | 1 | 0 | 0 | 0 | 0 | 0 | 0 | 0 | 0 | 0 | 0 | 0 | 0 |
| 2015 | Ptatscheck | 51.8313 | 12.1088 | temperate | 0 | 1 | 0 | 1 | 1 | 0 | 0 | 0 | 0 | 0 | 0 | 0 | 0 | 0 | 1 | 1 | 0 |
| 2016 | Gossner | 51.0948 | 10.3873 | temperate | 1 | 0 | 0 | 1 | 1 | 0 | 0 | 0 | 0 | 0 | 0 | 0 | 0 | 0 | 0 | 0 | 0 |
| 2016 | Nicholas | 11.021642 | -85.474337 | tropical | 0 | 1 | 0 | 1 | 1 | 0 | 0 | 0 | 0 | 0 | 0 | 1 | 0 | 0 | 0 | 1 | 0 |
| 2016 | Petermann | 51.0948 | 10.3873 | temperate | 0 | 1 | 1 | 0 | 1 | 0 | 0 | 0 | 0 | 0 | 0 | 0 | 0 | 0 | 0 | 0 | 0 |
| 2017 | Gossner | 49.859155 | 10.484266 | temperate | 1 | 0 | 0 | 1 | 1 | 0 | 1 | 0 | 0 | 0 | 0 | 0 | 0 | 0 | 0 | 0 | 0 |
| 2017 | Magyar | 47.5138 | 19.0121 | temperate | 1 | 0 | 0 | 1 | 0 | 0 | 0 | 0 | 0 | 0 | 0 | 0 | 0 | 1 | 0 | 0 | 0 |
| 2017 | Petermann | 47.8112 | 13.033229 | temperate | 1 | 0 | 0 | 1 | 1 | 0 | 1 | 0 | 1 | 0 | 0 | 0 | 0 | 0 | 1 | 0 | 0 |
| 2018 | Gossner b | 48.2695 | 23.6207 | temperate | 1 | 0 | 0 | 1 | 1 | 0 | 0 | 0 | 0 | 0 | 0 | 0 | 0 | 0 | 0 | 0 | 0 |
| 2018 | Gossner a | 48.2695 | 23.6207 | temperate | 0 | 1 | 0 | 1 | 1 | 0 | 0 | 0 | 0 | 0 | 0 | 0 | 0 | 0 | 0 | 0 | 0 |
| 2018 | Gossner c | 48.4 | 11.71 | temperate | 1 | 0 | 0 | 1 | 1 | 0 | 0 | 0 | 0 | 0 | 0 | 0 | 0 | 0 | 0 | 0 | 0 |
| 2018 | Petermann | 47.8112 | 13.033229 | temperate | 1 | 0 | 0 | 1 | 1 | 0 | 1 | 0 | 1 | 0 | 0 | 0 | 0 | 0 | 1 | 0 | 0 |
| 2018 | Yoshida | 36.35 | 139.6 | temperate | 0 | 1 | 1 | 0 | 1 | 0 | 0 | 0 | 0 | 0 | 0 | 0 | 0 | 0 | 1 | 0 | 0 |
| 2019 | Petermann | 47.8112 | 13.033229 | temperate | 1 | 0 | 0 | 1 | 1 | 0 | 1 | 0 | 1 | 0 | 0 | 0 | 0 | 0 | 1 | 0 | 0 |
| 2020 | Petermann | 51.0948 | 10.3873 | temperate | 1 | 0 | 0 | 1 | 1 | 0 | 0 | 0 | 0 | 0 | 0 | 0 | 1 | 0 | 1 | 0 | 0 |
| 2020 | Ranasinghe | 7.2662 | 80.6269 | tropical | 1 | 1 | 0 | 1 | 0 | 0 | 0 | 0 | 0 | 0 | 0 | 1 | 0 | 0 | 0 | 1 | 0 |
| 2020 | Shelomi | 24.759694 | 121.590306 | subtropical | 1 | 0 | 0 | 1 | 1 | 0 | 0 | 0 | 0 | 0 | 1 | 0 | 1 | 0 | 0 | 0 | 0 |
| 2021 | Rivett | 51.4082 | 0.6452 | temperate | 1 | 0 | 0 | 1 | 0 | 0 | 0 | 0 | 0 | 0 | 0 | 0 | 1 | 0 | 0 | 0 | 0 |
| 2021 | Snoeks | 8.75382 | -3.77325 | tropical | 1 | 0 | 0 | 1 | 1 | 0 | 1 | 0 | 0 | 0 | 0 | 0 | 0 | 0 | 0 | 0 | 0 |
| 2021 | Caragata | 18.481016 | -66.400537 | tropical | 1 | 0 | 0 | 1 | 0 | 0 | 0 | 0 | 1 | 0 | 0 | 0 | 0 | 0 | 0 | 0 | 0 |

**Table S2:** Results from mixed-effects meta-analyses on A) the effect of tree-hole size on organism abundance, B) the effect of tree-hole size on organism richness, C) the effect of detritus amount on organism abundance and D) the effect of detritus amount on organism richness. Upper/Lower CI = Upper/lower bound of 95% confidence interval. P-values<0.05 are printed in bold.

| **A) Size effect on abundance** | |  |  |  |  |  |
| --- | --- | --- | --- | --- | --- | --- |
|  | **Estimate** | **St. error.** | **Z** | **P** | **Lower CI** | **Upper CI** |
| **Intercept** | -1.0042 | 2.8789 | -0.3488 | 0.7272 | -6.6468 | 4.6383 |
| **Absolute latitude** | 0.013 | 0.0448 | 0.29 | 0.7718 | -0.0748 | 0.1008 |
| **Longitude** | -0.0016 | 0.004 | -0.3868 | 0.6989 | -0.0095 | 0.0063 |
| **Ann. mean temp.** | 0.0712 | 0.0581 | 1.2267 | 0.2199 | -0.0426 | 0.185 |
| **Ann. precipitation** | -0.0003 | 0.0005 | -0.6321 | 0.5273 | -0.0012 | 0.0006 |
| **Artificial vs. natural** | 0.2026 | 0.1123 | 1.8043 | 0.0712 | -0.0175 | 0.4226 |
| **Insects vs. others** | -0.1382 | 0.1321 | -1.046 | 0.2956 | -0.3971 | 0.1207 |
|  |  |  |  |  |  |  |
| **B) Size effect on richness** | |  |  |  |  |  |
|  | **Estimate** | **St. error.** | **Z** | **P** | **Lower CI** | **Upper CI** |
| **Intercept** | -3.5503 | 2.711 | -1.3096 | 0.1903 | -8.8637 | 1.7631 |
| **Absolute latitude** | 0.0613 | 0.0414 | 1.4805 | 0.1387 | -0.0198 | 0.1424 |
| **Longitude** | -0.0031 | 0.003 | -1.0225 | 0.3066 | -0.0089 | 0.0028 |
| **Ann. mean temp.** | 0.1239 | 0.0527 | 2.3526 | **0.0186** | 0.0207 | 0.2271 |
| **Ann. precipitation** | 0.0002 | 0.0005 | 0.3872 | 0.6986 | -0.0007 | 0.0011 |
| **Artificial vs. natural** | -0.2318 | 0.2121 | -1.0931 | 0.2744 | -0.6474 | 0.1838 |
| **Insects vs. others** | -0.195 | 0.1354 | -1.4395 | 0.15 | -0.4604 | 0.0705 |
|  |  |  |  |  |  |  |
| **C) Detritus effect on abundance** | | |  |  |  |  |
|  | **Estimate** | **St. error.** | **Z** | **P** | **Lower CI** | **Upper CI** |
| **Intercept** | -1.0467 | 1.578 | -0.6633 | 0.5071 | -4.1394 | 2.046 |
| **Absolute latitude** | 0.0226 | 0.0234 | 0.9662 | 0.334 | -0.0233 | 0.0685 |
| **Longitude** | -0.006 | 0.0024 | -2.5079 | **0.0121** | -0.0108 | -0.0013 |
| **Ann. mean temp.** | -0.0253 | 0.0307 | -0.8248 | 0.4095 | -0.0855 | 0.0348 |
| **Ann. precipitation** | 0.0004 | 0.0005 | 0.8432 | 0.3991 | -0.0005 | 0.0013 |
| **Artificial vs. natural** | 0.2643 | 0.0863 | 3.0614 | **0.0022** | 0.0951 | 0.4335 |
| **Insects vs. others** | 0.0595 | 0.0797 | 0.7473 | 0.4549 | -0.0966 | 0.2157 |
|  |  |  |  |  |  |  |
| **D) Detritus effect on richness** | |  |  |  |  |  |
|  | **Estimate** | **St. error.** | **Z** | **P** | **Lower CI** | **Upper CI** |
| **Intercept** | -7.0353 | 1.6351 | -4.3028 | **<0.0001** | -10.24 | -3.8307 |
| **Absolute latitude** | 0.1099 | 0.0245 | 4.4833 | **<0.0001** | 0.0619 | 0.1579 |
| **Longitude** | -0.0058 | 0.0025 | -2.3021 | **0.0213** | -0.0107 | -0.0009 |
| **Ann. mean temp.** | 0.0817 | 0.0319 | 2.5608 | **0.0104** | 0.0192 | 0.1442 |
| **Ann. precipitation** | 0.0015 | 0.0005 | 3.2658 | **0.0011** | 0.0006 | 0.0024 |
| **Artificial vs. natural** | 0.0938 | 0.0887 | 1.0573 | 0.2904 | -0.0801 | 0.2677 |
| **Insects vs. others** | -0.1629 | 0.124 | -1.3139 | 0.1889 | -0.4058 | 0.0801 |

**References Appendix:**

Ager, D., S. Evans, H. Li, A. K. Lilley, and C. J. Van Der Gast. 2010. Anthropogenic disturbance affects the structure of bacterial communities. Environmental Microbiology 12:670-678.

Barrera, R. 1996. Species concurrence and the structure of a community of aquatic insects in tree holes. Journal of Vector Ecology 21:66-80.

Bell, T. 2010. Experimental tests of the bacterial distance-decay relationship. Isme Journal 4:1357-1365.

Bell, T., D. Ager, J.-I. Song, J. A. Newman, I. P. Thompson, A. K. Lilley, and C. J. van der Gast. 2005. Larger Islands House More Bacterial Taxa. Science 308:1884-1884.

Blakely, T. J., and R. K. Didham. 2010. Disentangling the mechanistic drivers of ecosystem-size effects on species diversity. Journal of Animal Ecology 79:1204-1214.

Blakely, T. J., J. S. Harding, and R. K. Didham. 2012. Distinctive aquatic assemblages in water-filled tree holes: a novel component of freshwater biodiversity in New Zealand temperate rainforests. Insect Conservation and Diversity 5:202-212.

Büermann, S. 2015. The influence of urbanity and other environmental factors on the community structures of larval macroinvertebrates living in urban temporary water bodies. BSc Thesis. Freie Universität Berlin.

Caragata, E. P., L. M. Otero, C. V. Tikhe, R. Barrera, and G. Dimopoulos. 2021. Microbial Diversity of Adult Aedes aegypti and Water Collected from Different Mosquito Aquatic Habitats in Puerto Rico. Microbial Ecology in Health and Disease.

Copeland, R. 1989. The Insects of Treeholes of Northern Indiana With Special Reference to Megaselia scalaris (Diptera: Phoridae) and Spilomyia longicornis (Diptera: Syrphidae). Great Lakes Entomologist 22:4.

Copeland, R. S., M. De Meyer, and G. E. Rotheray. 1999. First record of phytotelmata and aquatic insects from the Cycadales, with a description of the puparium of Senaspis haemorrhoa Gerstaecker (Diptera: Syrphidae). African Entomology 7:157-160.

Cumberlidge, N., D. Fenolio, xe, B, E. W. Mark, and J. Stout. 2005. Tree-Climbing Crabs (Potamonautidae and Sesarmidae) from Phytotelmic Microhabitats in Rainforest Canopy in Madagascar. Journal of Crustacean Biology 25:302-308.

Devetter, M. 2004. Invertebrate fauna of treeholes in relation to some habitat conditions in southern Bohemia (Czech Republic). Acta Societatis Zoologicae Bohemicae 68:161-168.

Gönczöl, J., and Á. Révay. 2003. Treehole fungal communities: Aquatic, aero-aquatic and dematiaceous hyphomycetes. Fungal Diversity 12.

Gossner, M. 2017. Invertebrate communities of tree holes in Steigerwald, Germany. Unpublished data set.

Gossner, M. 2018a. Invertebrate communities of artificial tree holes in Ukraine. Unpublished data set.

Gossner, M. 2018b. Invertebrate communities of natural tree holes in Ukraine. Unpublished data set.

Gossner, M. 2018c. A three year study of the phenology of insect larvae (Coleoptera, Diptera) in water-filled tree holes in the canopy of a beech tree. European Journal of Entomology 115:524-534.

Gossner, M., P. Lade, A. Schober, N. Sichardt, T. Kahl, J. Bauhus, W. W. Weisser, and J. S. Petermann. 2016. Effects of management on aquatic tree-hole communities in temperate forests are mediated by detritus amount and water chemistry. Journal of Animal Ecology 96:428–439.

Green, D. 1990. A study of the faunas of water-filled tree-holes and memorial vases. The Vasculum 75:27-39.

Harlan, N. P., and C. J. Paradise. 2006. Do habitat size and shape modify abiotic factors and communities in artificial treeholes? Comm Ecology 7.

Heaps, J. W. 1981. Insect colonization of drilled tree holes. Entomological News 92:106-110.

Jenkins, B., and R. L. Kitching. 1990. The ecology of water-filled treeholes in Australian rainforests: Food web reassembly as a measure of community recovery after disturbance. Australian Journal of Ecology 15:199-205.

Jenkins, B., R. L. Kitching, and S. L. Pimm. 1992. Productivity, Disturbance and Food Web Structure at a Local Spatial Scale in Experimental Container Habitats. Oikos 65:249-255.

Karamchand, K. S., and K. R. Sridhar. 2008. Water-borne conidial fungi inhabiting tree holes of the west coast and Western Ghats of India. Czech Mycology 60:63-74.

Kaufman, M. G., S. Chen, and E. D. Walker. 2008. Leaf-Associated Bacterial and Fungal Taxa Shifts in Response to Larvae of the Tree Hole Mosquito, Ochlerotatus triseriatus. Microbial Ecology 55:673-684.

Keilin, D. 1927. Fauna of a Horse-Chestnut Tree (Aesculus hippocastanum). Dipterous Larvae and their Parasites. Parasitology 19:368-374.

Khazan, E. S., E. G. Bright, and J. E. Beyer. 2015. Land management impacts on tree hole invertebrate communities in a Neotropical rainforest. Journal of Insect Conservation 19:681-690.

Kitching, R., and A. Orr. 1996. The foodweb from water-filled treeholes in Kuala Belalong, Brunei. The Raffles Bulletin of Zoology 44:405-413.

Kitching, R. L. 1969. A preliminary note of the fauna of water-filled tree-holes. The Entomologist.

Kitching, R. L. 1987a. A preliminary account of the metazoan food webs in phytotelmata from Sulawesi, Indonesia. Malayan Nature Journal 41:1-12.

Kitching, R. L. 1987b. Spatial and temporal variation in food webs in water-filled treeholes. Oikos 48.

Kitching, R. L. 1990. Foodwebs from phytotelmata in Madang, Papua New Guinea. Entomologist 109:153-164.

Kitching, R. L., and C. Callaghan. 1982. The fauna of water-filled tree holes in box forest in south-east Queensland. Australian Entomological Magazine 8:61-70.

Lackey, J. B. 1940. The microscopic flora and fauna of tree holes. The Ohio Journal of Science 40:186-192.

Magyar, D., M. Vass, and G. Oros. 2017. Dendrotelmata (Water-Filled Tree Holes) as Fungal Hotspots - A Long Term Study. Cryptogamie, Mycologie 38:55-66.

Mayer, K. 1938. Zur Kenntnis der Buchenhöhlenfauna. Archiv für Hydobiologie 33:388-400.

Nicholas, A. 2016. Forest fragmentation changes macroinvertebrate community composition in tropical tree holes University of British Columbia, Vancouver, Canada.

Nishadh, K. A., and K. S. A. Das. 2012. Metazoan communities in tree-hole aquatic habitat of Silent Valley National Park and New Amarambalam Reserved Forest of the Western Ghats, India.

Paradise, C. J. 1998. Colonization and development of insects in simulated treehole habitats with distinct resource and pH regimes Ecoscience 5:39-45.

Paradise, C. J. 2004. Relationship of water and leaf litter variability to insects inhabiting treeholes. Journal of the North American Benthological Society 23:793-805.

Paradise, C. J., J. D. Blue, J. Q. Burkhart, J. Goldberg, L. Harshaw, K. D. Hawkins, B. Kegan, T. Krentz, L. Smith, and S. Villalpando. 2008. Local and regional factors influence the structure of treehole metacommunities. BMC Ecology 8:1-16.

Paradise, C. J., and W. A. Dunson. 1997. Effects of Water Cations on Treehole Insect Communities. Annals of the Entomological Society of America 90:798-805.

Pavisic, V. 1938. Über die Ökologie der Baumhöhlenmückenlarven in Jugoslavien. Archiv für Hydobiologie 33:700-705.

Petermann, J. S. 2017. Invertebrate communities of tree holes in Salzburg, Austria. Unpublished data set.

Petermann, J. S. 2018. Invertebrate communities of tree holes in Salzburg, Austria. Unpublished data set.

Petermann, J. S. 2019. Invertebrate communities of tree holes in Salzburg, Austria. Unpublished data set.

Petermann, J. S., A. L. Roberts, C. Hemmerling, F. Bajerski, J. Pascual, J. Overmann, W. W. Weisser, L. Ruess, and M. Gossner. 2020. Direct and indirect effects of forest management on tree-hole inhabiting aquatic organisms and their functional traits. Science of the Total Environment 704:135418.

Petermann, J. S., A. Rohland, N. Sichardt, P. Lade, B. Guidetti, W. W. Weisser, and M. Gossner. 2016. Forest management intensity affects aquatic communities in artificial tree holes. PloS ONE 11:e0155549.

Pimm, S. L., and R. L. Kitching. 1987. The Determinants of Food Chain Lengths. Oikos 50:302-307.

Ponnusamy, L., N. Xu, G. Stav, D. M. Wesson, C. Schal, and C. S. Apperson. 2008. Diversity of Bacterial Communities in Container Habitats of Mosquitoes. Microbial Ecology 56:593-603.

Ptatscheck, C., and W. Traunspurger. 2014. The meiofauna of artificial water-filled tree holes: colonization and bottom-up effects. Aquatic Ecology 48:285-295.

Ptatscheck, C., and W. Traunspurger. 2015. Meio- and Macrofaunal Communities in Artificial Water-Filled Tree Holes: Effects of Seasonality, Physical and Chemical Parameters, and Availability of Food Resources. PloS ONE 10:e0133447.

Ranasinghe, K., and D. Amarasinghe. 2020. Naturally Occurring Microbiota in Dengue Vector Mosquito Breeding Habitats and Their Use as Diet Organisms by Developing Larvae in the Kandy District, Sri Lanka. BioMed Research International 2020:5830604.

Rivett, D. W., S. B. Mombrikotb, H. S. Gweon, T. Bell, and C. van der Gast. 2021. Bacterial communities in larger islands have reduced temporal turnover. The ISME Journal 15:2947-2955.

Rohnert, U. 1950. Wassererfüllte Baumhöhlen und ihre Besiedlung: ein Beitrag zur Fauna dendrolimnetica. Archiv für Hydobiologie 44:472-518.

Sanchez, E., and J. Liria. 2009. Relative abundance and temporal variation of macroinvertebrates in a Venezuelan cloud forest habitat. International Journal of Tropical Insect Science 29:3-10.

Schmidl, J., P. Sulzer, and R. L. Kitching. 2008. The insect assemblage in water filled tree-holes in a European temperate deciduous forest: community composition reflects structural, trophic and physicochemical factors. Hydrobiologia 598:285-303.

Schulz, G., T. Siqueira, G. Stefan, and F. de Oliveira Roque. 2012. Passive and active dispersers respond similarly to environmental and spatial processes: an example from metacommunity dynamics of tree hole invertebrates. Fundamental and Applied Limnology / Archiv für Hydrobiologie 181:315-326.

Shelomi, M., and C.-T. Lin. 2020. Mosquito and bacterial diversity in Phytotelmata in northern Taiwan. International Journal of Tropical Insect Science.

Smith, L. M., J. D. Blue, J. Carlson, G. Metz, J. Haywood, D. Bush, and C. J. Paradise. 2009. Density-Dependent Predation of a Dominant Species does not Facilitate Increased Diversity in Treeholes. The Open Ecology Journal 2:91-99.

Snoeks, J. M., M. Driesen, S. Porembski, Á. Aristizábal-Botero, and B. Vanschoenwinkel. 2021. Contrasting biodiversity and food web structure of three temporary freshwater habitats in a tropical biodiversity hotspot. Aquatic Conservation: Marine and Freshwater Ecosystems 31:2603-2620.

Snow, W. E. 1958. Stratification of Arthropods in a Wet Stump Cavity. Ecology 39:83-88.

Sota, T. 1996. Effects of capacity on resource input and the aquatic metazoan community structure in phytotelmata. Researches on Population Ecology 38:65-73.

Sota, T. 1998. Microhabitat size distribution affects local difference in community structure: Metazoan communities in treeholes. Researches on Population Ecology 40:249-255.

Srivastava, D. S. 2005. Do local processes scale to global patterns? The role of drought and the species pool in determining treehole insect diversity. Oecologia 145:205-215.

Srivastava, D. S., and J. H. Lawton. 1998. Why more productive sites have more species: An experimental test of theory using tree-hole communities. American Naturalist 152:510-529.

Taylor, R., and R. M. Ewers. 2003. The invertebrate fauna inhabiting tree holes in a red beech (Nothofagus fusca) tree. 25.

Thienemann, A. 1934. Die Tierwelt der tropischen Pflanzengewasser. Archiv für Hydrobiologie Supplement 13:1-91.

Verdonschot, R. C. M., C. M. Febria, and D. D. Williams. 2008. Fluxes of dissolved organic carbon, other nutrients and microbial communities in a water-filled treehole ecosystem. Hydrobiologia 596:17-30.

von Brandt, A. 1934. Untersuchungen in Baumhöhlengewässern auf Fagus silvatica. Archiv für Hydobiologie 27:546-563.

Walker, E. D., M. G. Kaufman, and R. W. Merritt. 2010. An acute trophic cascade among microorganisms in the tree hole ecosystem following removal of omnivorous mosquito larvae. Community Ecology 11:171-178.

Woodward, F. I. 1988. The Aquatic Insect Communities of Tree Holes in Northern California Oak Woodlands. Bulletin of the Society of Vector Ecologists 13:221-234.

Yanoviak, S. P. 1999a. Community structure in water-filled tree holes of Panama: effects of hole height and size. Selbyana 20:106-115.

Yanoviak, S. P. 1999b. Effects of leaf litter species on macroinvertebrate community properties and mosquito yield in Neotropical tree hole microcosms. Oecologia 120:147-155.

Yanoviak, S. P. 2001a. Container color and location affect macroinvertebrate community structure in artificial treeholes in Panama. Florida Entomologist 84:265-271.

Yanoviak, S. P. 2001b. The Macrofauna of Water-filled Tree Holes on Barro Colorado Island, Panama. Biotropica 33:110-120.

Yanoviak, S. P. 2001c. Predation, resource availability, and community structure in Neotropical water-filled tree holes. Oecologia 126:125-133.

Yanoviak, S. P., L. P. Lounibos, and S. C. Weaver. 2006. Land use affects macroinvertebrate community composition in phytotelmata in the Peruvian Amazon. Annals of the Entomological Society of America 99:1172-1181.

Yee, D. A., and S. A. Juliano. 2007. Abundance Matters: A Field Experiment Testing the More Individuals Hypothesis for Richness-Productivity Relationships. Oecologia 153:153-162.

Yee, D. A., and S. A. Juliano. 2012. Concurrent effects of resource pulse amount, type, and frequency on community and population properties of consumers in detritus-based systems. Oecologia 169:511-522.

Yee, D. A., S. H. Yee, J. M. Kneitel, and S. A. Juliano. 2007. Richness-productivity relationships between trophic levels in a detritus-based system: significance of abundance and trophic linkage. Oecologia 154:377-385.

Yoshida, T., Y. Ban, and A. Nakamura. 2018. Vertical stratification of invertebrate assemblages in water-filled treeholes of a temperate deciduous forest. Basic and Applied Ecology 27:61-70.
